# The Comparative Anatomy of the Metatarsal Foot Pad in Eight Species of Birds of Prey and Owls with Regard to the Development of Pododermatitis

**DOI:** 10.3390/vetsci12050498

**Published:** 2025-05-19

**Authors:** Rebekka Schwehn, Elisabeth Engelke, Christian Seiler, Dominik Fischer, Hermann Seifert, Christiane Pfarrer, Michael Fehr, Marko Legler

**Affiliations:** 1 Department of Small Mammal, Reptile and Avian Medicine and Surgery, University of Veterinary Medicine Hannover, Foundation, Bünteweg 9, 30559 Hannover, Germany; michael.fehr.ir@tiho-hannover.de (M.F.); marko.legler@tiho-hannover.de (M.L.); 2 Institute for Anatomy, University of Veterinary Medicine Hannover, Foundation, Bischofsholer Damm 15, 30173 Hannover, Germany; elisabeth.engelke@tiho-hannover.de (E.E.); christiane.pfarrer@tiho-hannover.de (C.P.); 3 Institute for General Radiology and Medical Physics, University of Veterinary Medicine Hannover, Foundation, Bischofsholer Damm 15, 30173 Hannover, Germany; 4 Clinic for Birds, Reptiles, Amphibians and Fish, Justus Liebig University Giessen, Frankfurter Str. 114, 35392 Giessen, Germany; fischer@zoo-wuppertal.de; 5 Zoo Wuppertal, Hubertusallee 30, 42117 Wuppertal, Germany

**Keywords:** avian anatomy, fat pad, blood vessel topography, arteriography, venography, contrast micro-computed tomography scan, pelvic limb, pododermatitis, bumblefoot, raptor

## Abstract

Pododermatitis (bumblefoot) occurs commonly in birds of prey and owls and shows species-specific differences in its prevalence, especially between falcons and hawks. The development of the disease is particularly associated with circulatory disorders of the feet. To compare the blood vessel supply of the foot sole, especially the metatarsal foot pad, macroscopic dissections including intravascular injection of latex, contrast µCT scans with barium sulphate, corrosion casts (epoxy resin), and histological examinations of the skin were performed in eight avian species. The main supplying artery of the metatarsal pad, the pulvinar artery, originated from the medial digital artery of the first toe. This main pulvinar artery showed a rather vertical course towards the sole surface, subsequently encircling the metatarsal pad like a basket in falcons and owls, whereas a rather horizontal straight course was observed in northern goshawks and common buzzards. Organized fat tissue was present in the subcutis as the basis for the metatarsal pad only in falcons and owls. The dermis of the metatarsal pad showed a well-developed papillary layer and prominent dermal and subdermal vascular networks in all species examined. The observed differences were discussed regarding both the species-specific prevalence and the etiology of bumblefoot.

## Simple Summary

Pododermatitis (bumblefoot) is the devitalization and inflammation of the skin of the foot sole in the region of the metatarsal foot pad. The disease appears to be related to circulatory disorders of the feet, and some species of birds of prey and owls are more frequently affected than others. The purpose of the study was to demonstrate the supplying blood vessels of the metatarsal pad and compare them between different species. In order to make the vessels visible in the dissected specimens, they were filled with colored injection material. In addition, µCT 3D reconstructions and microscopic evaluations of histological sections of the skin were made. The results showed differences between the examined species with regard to the course of the main supplying artery of the metatarsal pad. In addition, the structure of the metatarsal pad varied between species in the presence and amount of fat tissue. These differences may play a role in the development of pododermatitis and thus be associated with the different vulnerability of the species.

## 1. Introduction

Pododermatitis is a common disease of the foot sole in captive birds of prey that affects the region of the metatarsal foot pad and is also known as bumblefoot [1,2,3,4]. Lierz [5] described an incidence of 10.1% in 4193 falcons during their first year of falconry use in Abu Dhabi, United Arab Emirates. Müller [6,7] reported the occurrence of pododermatitis in 27.6% of 549 falcons admitted to veterinary clinics in Dubai and Abu Dhabi, United Arab Emirates. Bumblefoot is also described in owls, but the literature is rather sparse [8,9]. The progress of the disease is characterized by the devitalization of the epithelium on the plantar surface of the foot, followed by bacterial invasion and deep tissue inflammation up to tissue destruction and the total loss of the pedal function [3,10,11]. Thus, bumblefoot is graded into different stages based on the course, severity, and chronicity of the disease [11,12,13].

Husbandry conditions such as unsuitable perches, the birds being overweight, and a lack of physical activity are discussed as possible causes for this disease [2,14,15,16]. Studies show that the incidence of pododermatitis is lower in falcons that are exercised twice a day than in those that are exercised only once a day [5,6,7]. Problems occur more commonly in trained falcons when physical activity is abruptly ended—for example, at the end of the hunting season—than in those whose training is gradually reduced [3,5,6,7,17]. Heidenreich [3,17] compares this to highly trained athletes, who show severe cardiovascular complications and an excess cardiac capacity if they abruptly decrease their level of physical activity. In birds of prey, the pathogenesis might be similar, resulting in dependent edema, particularly in the feet, and secondary ischemic pressure-induced necrosis [3,14,17]. These observations are consistent with studies on falcons showing that during training, the blood flow to the skin of the feet is significantly higher, which is reflected in an increased foot skin temperature [5,18]. Thus, training seems to be effective in preventing and supporting recovery from ischemic necrosis of the skin of the feet [18]. In conclusion, the etiology of bumblefoot appears to be closely related to circulatory disorders of the feet [2,3,5].

There are species-specific differences in the prevalence of the development of pododermatitis: Compared to hawks and owls, falcons are affected more frequently or more severely [1,3,14,19,20]. In contrast to the accipiters, which tend to hunt at short distances, falcons are mainly “long-distance hunters” [3,17]. The biological differences between species in flight activity and hunting behavior might result in a greater discrepancy between the cardiovascular fitness level of an active and an inactive falcon compared to an active and an inactive hawk [3,14,17]. This might be a reason for a higher susceptibility for the development of circulatory disorders in falcons [3,14,17].

The species-specific prevalence of pododermatitis led us to the question whether there are anatomical differences in the blood vessel topography of the foot that might play a role in the susceptibility for the development of the disease. Former studies on the general vasculature of the feet in birds of prey and owls did not go into sufficient detail concerning the blood vessel topography of the foot sole [14,21,22,23,24,25,26]. In a previous study, we were already able to compare and visualize in detail the course of metatarsal and digital blood vessels in eight species of birds of prey and owls [27]. Nevertheless, a description of the interspecific variations in the blood vessel supply of the foot sole in birds of prey and owls was still missing.

The purpose of this study was to describe the skin layers, i.e., epidermis, dermis, and subcutis, as well as the vasculature of the metatarsal foot pad, and, in comparison, the proximal digital foot pad of the second toe and the area cranial to the metatarsal pad. We focused on interspecific anatomical differences in Falconiformes, Accipitriformes, and Strigiformes in order to identify a potential link with the bumblefoot prevalence described above.

## 2. Materials and Methods

Examinations were carried out on carcasses of fully grown specimens of eight avian species: northern goshawk (*Accipiter gentilis*), common buzzard (*Buteo buteo*), peregrine falcon (*Falco peregrinus*), hybrid gyrfalcon × saker falcon (gyr–saker falcon; *Falco rusticolus* × *Falco cherrug*), common kestrel (*Falco tinnunculus*), Eurasian eagle-owl (*Bubo bubo*), long-eared owl (*Asio otus*), and barn owl (*Tyto alba*). Table 1 shows the number of the investigated specimens per species, sex, and research method.

The gyr–saker falcons used in this study were captive-bred individuals that had previously been serving as a control group in an earlier research project. The animal experiment and all necessary measures of this former study had been approved by the Animal Protection Commission of the Regierungspräsidium Giessen, Germany, with the approval number GI18/9 No. 69/2011. Cadavers were made available for the present study after completion of this former project.

The remaining specimens were wild birds that had been admitted as patients to the Department of Small Mammal, Reptile and Avian Medicine and Surgery of the University of Veterinary Medicine Hannover, Germany. The birds had been euthanized for animal welfare purposes due to serious injuries that would have made a later release into the wild impossible [28,29]. All related procedures were conducted in accordance with the German animal welfare law (Tierschutzgesetz §4, §7, and §7a) as well as the Directive of the European Parliament and of the Council for the Protection of Animals Used for Experimental and other Scientific Purposes (2010/63/EU). Accordingly (Tierschutzgesetz §7), no explicit permission was needed to carry out this study as no medical procedures or experiments were performed while the animals were alive. This procedure was approved by the University’s Animal Welfare Officer, confirmation TVO-2017-V-61.

The macroscopic surface relief of the skin of the foot sole was visually examined in 19 to 24 specimens per species (Table 1) by simply stretching out the toes. In addition, longitudinal sections of the feet were made centered through toe I, the metatarsal foot pad, and toe III on frozen specimens (two feet per species, all specimens male) using a bandsaw (MICRO bandsaw MBS 240/E, PROXXON S.A., Wecker, Luxembourg) to be able to better demonstrate the localization and appearance of the metatarsal pad macroscopically. The specimens were photographed for the purpose of documentation (Sony SLT-A58 digital camera and Sony SAL 18–55 mm F3.5-5.6 lens, Sony Corporation, Minato, Tokyo, Japan).

For the histological examination, six birds of each species were selected (Table 1). Carcasses from recently deceased animals were used, except for the gyr–saker falcons, which had been frozen after death at −20 °C until examination and were thawed for taking histological samples. For the preparation of the histological sections, the skin—consisting of epidermis, dermis, and subcutis—of the sole of the foot was dissected exactly along the flexor tendons underneath. Specimens were fixed with formalin (Roti^®^-Histofix 4%, phosphate buffered, Carl Roth, Karlsruhe, Germany) for at least seven days and then transferred to 70% ethanol and routinely processed. Subsequently, the specimens were embedded in paraffin (Paraplast Bulk REF 36602012, Leica Microsystems CMS GmbH, Wetzlar, Germany). Sections (2–3 μm thick) were prepared from three different localizations of the sole: the metatarsal foot pad, the interpulvinar area directly cranial to the metatarsal pad, and the proximal digital foot pad of the second toe. For each bird, cross-sections of the skin were made on one foot and longitudinal sections on the other foot. The skin was cut at the same locations in all specimens to minimize variations due to dissection techniques. Sections were stained with hematoxylin–eosin (HE) or Masson-Goldner trichrome. Photographs were taken with digital cameras (Olympus SC50, Olympus Corporation, Tokyo, Japan; Leica DFC310 FX, Leica Microsystems CMS GmbH, Wetzlar, Germany) attached to light microscopes (ZEISS Axioskop, Carl Zeiss AG, Jena, Germany; Leica DM6000 B, Leica Microsystems CMS GmbH, Wetzlar, Germany) and imaging software (Olympus cellSens Standard 2.2, Olympus Corporation, Tokyo, Japan; Leica Application Suite Version 3.4.1, Leica Microsystems CMS GmbH, Wetzlar, Germany). For the classification of the subcutis into different categories in regard to the amount and arrangement of fat tissue (see Table 2), the histological sections were evaluated descriptively.

Five to nine birds were used (Table 1) to create the corrosion casts for each species studied. All cadavers had been stored at −20 °C and were thawed prior to examination. The sternum was removed to allow access to the heart, and an amount of 20 to 100 mL epoxy resin (Biodur^®^ E20 Plus, Biodur^®^ Products GmbH, Heidelberg, Germany), depending on the size of the bird, was injected under manual pressure into the descending aorta. In this way, the entire blood vessel system of the caudal part of the body, including the feet, was filled with epoxy resin. In case of insufficient filling of the pedal blood vessels via the aorta, the ischiadic artery was used for a second attempt. The feet were placed on a plexiglass plate, and the spread toes were fixed with a thin plastic-coated wire. Specimens were kept at a temperature of 6–8 °C for one week to harden. Subsequently, the feet were severed and then macerated for two weeks to remove all soft tissues and expose the casts of the pedal vasculature including the blood vessel networks of the skin. The specimens were photographed for the purpose of documentation (Sony α7 III digital camera, Sony Corporation, Minato, Tokyo, Japan with Sigma 105 mm F2,8 Macro lens, SIGMA (Deutschland) GmbH, Rödermark, Germany). In addition, photographs were taken with a digital camera (ColorView Illu, Olympus Corporation, Tokyo, Japan) via a stereomicroscope (Stemi SV11, Carl Zeiss AG, Jena, Germany).

For macroscopic examination, the same specimens were used in this study that had been examined for the purposes of our previous study [27]. Latex injections and dissections as well as contrast micro-computed tomography (µCT) scans were performed. Three to seven birds per species and examination method were used (Table 1). All carcasses had been stored at −20 °C prior to examination. Subsequently, the specimens were thawed, and an incision was made on the medial side of the femur to access the major blood vessels of the hind limb. Colored latex (60%, Wurfbain Nordmann GmbH, Hamburg, Germany; color: Alpina Voll- und Abtönfarbe, Alpina Farben, Ober-Ramstadt, Germany) was injected to display the pedal blood vessels, with red latex used for the arteries and blue latex for the veins. Depending on the size of the bird, 0.4 to 3 mL of latex was injected under manual pressure either into the ischiadic artery for the arteries or into the external iliac vein for the veins. The access to the veins was retrograde, and, compared to the arteries, an increased manual pressure was necessary to fully fill the veins. If the pedal veins were insufficiently filled via the external iliac vein, the medial plantar metatarsal vein [27] was used for a second attempt, excluding very small species. However, in comparison to the pulvinar arteries, the number of pulvinar veins filled with injection material was smaller. The specimens were stored in plastic bags to prevent them from drying out and kept at 6–8 °C for seven days to allow the latex to harden. Afterward, the skin and tendons of the toes were removed to expose the course of the latex-filled digital blood vessels and their pulvinar branches supplying the metatarsal pad. Photographs were taken for documentation purposes using a Sony SLT-A58 digital camera and Sony SAL 18–55 mm F3.5–5.6 lens (Sony Corporation, Minato, Tokyo, Japan).

For contrast µCT scans, 2 to 20 mL barium sulphate (Barilux^®^ suspension, Sanochemia Diagnostics Deutschland GmbH, Neuss, Germany) depending on the size of the bird was injected into the ischiadic artery; the success of the filling was checked radiographically. In this way, the arteries as well as the veins of the foot were filled with contrast medium via arterio-venous anastomoses. To completely fill the vessels, a prolonged manual pressure was needed, which often resulted in leakage of contrast medium into the muscles of the femur and the tibiotarsus. µCT scans of the distal end of the tarsometatarsus and of the toes were conducted using an XtremeCT (Scanco Medical AG, Brüttisellen, Switzerland) with a fixed tube voltage of 60 kV. The resolution was set to 41 µm, the integration time to 700 ms. The resulting data were analyzed, and 3D reconstruction was generated using the Thermo Scientific™ Amira™ 3D visualization and analysis software (version 6.4.0, Thermo Fisher Scientific Inc., Waltham, MA, USA).

## 3. Results

**Figure 1:**
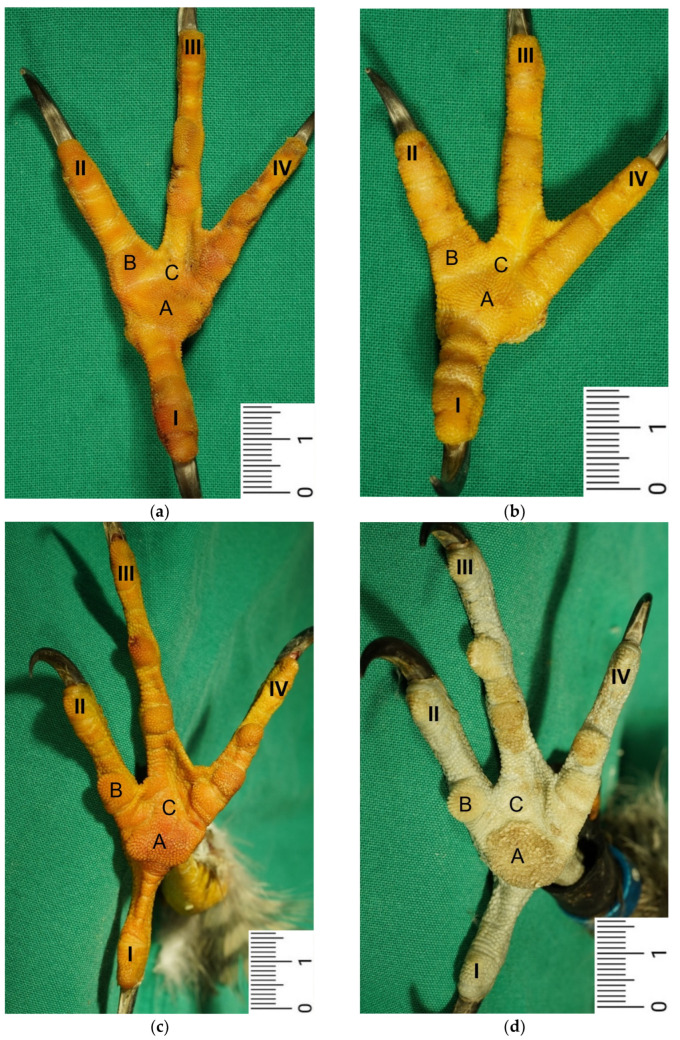

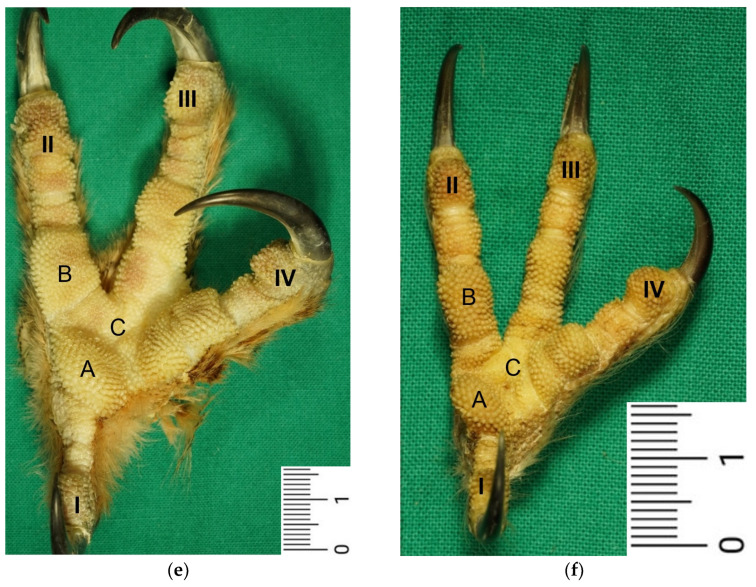
Skin of the foot sole in northern goshawk (**a**), common buzzard (**b**), peregrine falcon (**c**), gyr–saker falcon (**d**), Eurasian eagle-owl (**e**), and barn owl (**f**). Left foot, plantar aspect; A―metatarsal foot pad; B―proximal digital foot pad of the second toe; C―interpulvinar area cranial to the metatarsal foot pad; I–IV―1st to 4th toe.

**Figure 2:**
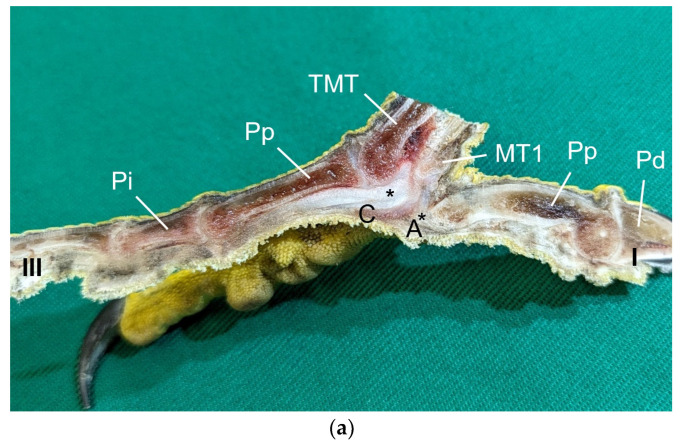

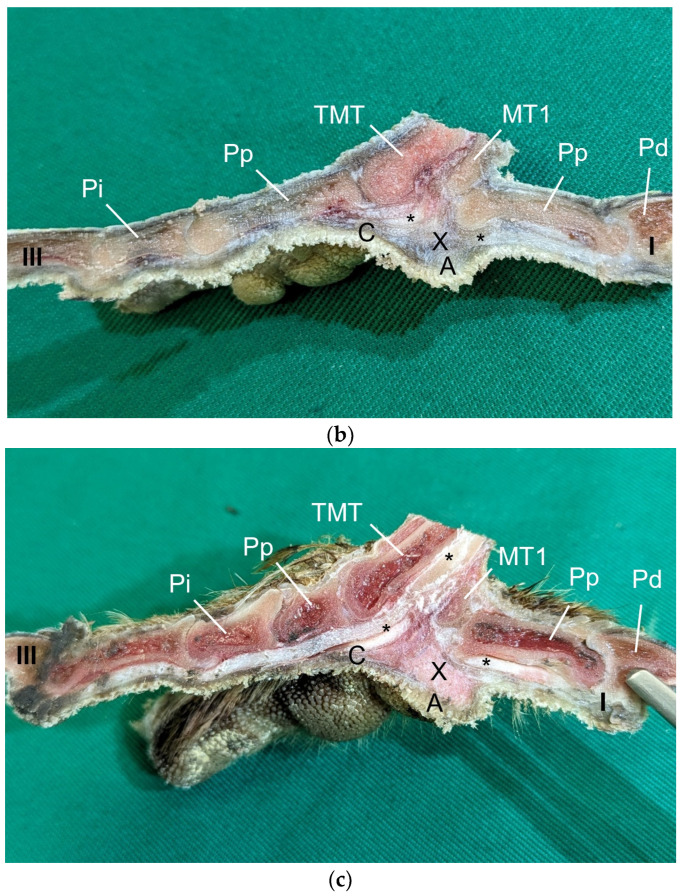
Longitudinal section through the right foot, centered through toe I, the metatarsal foot pad, and toe III in northern goshawk (**a**), gyr–saker falcon (**b**), and Eurasian eagle-owl (**c**). A―metatarsal foot pad; C―interpulvinar area cranial to the metatarsal foot pad; X―loose white to pinkish or yellowish fat tissue; *―flexor tendons; TMT―tarsometatarsus; MT1―first metatarsal bone; Pp―proximal phalanx of toes I and III; Pi―intermediate phalanx of toe III; Pd―distal phalanx of toe I; I―1st toe; III―3rd toe.

Macroscopically, the relief of the skin of the foot sole differed between the species studied. In common buzzards and northern goshawks, the metatarsal and digital foot pads could not be clearly distinguished from the surrounding skin; the plantar aspect of the foot seemed rather flat. In contrast, in the examined falcons and owls, the metatarsal and digital pads were more raised above the surface and could be differentiated more clearly from the interpulvinar skin areas (Figure 1). Longitudinal sections centered through the metatarsal pad confirmed that the skin was more protrusive in this region in falcons and owls; the sections displayed loose white to pinkish or yellowish tissue that macroscopically looked like fat tissue and was located between the flexor tendons and the cutis, i.e., the dermis and epidermis, of the metatarsal pad (Figure 2). In comparison, in common buzzards and northern goshawks, the longitudinal sections showed a rather flat metatarsal pad region and no macroscopically visible fat tissue between the flexor tendons and the cutis (Figure 2).

### 3.1. Skin Layers of the Foot Sole Including Vascular Networks

**Figure 3:**
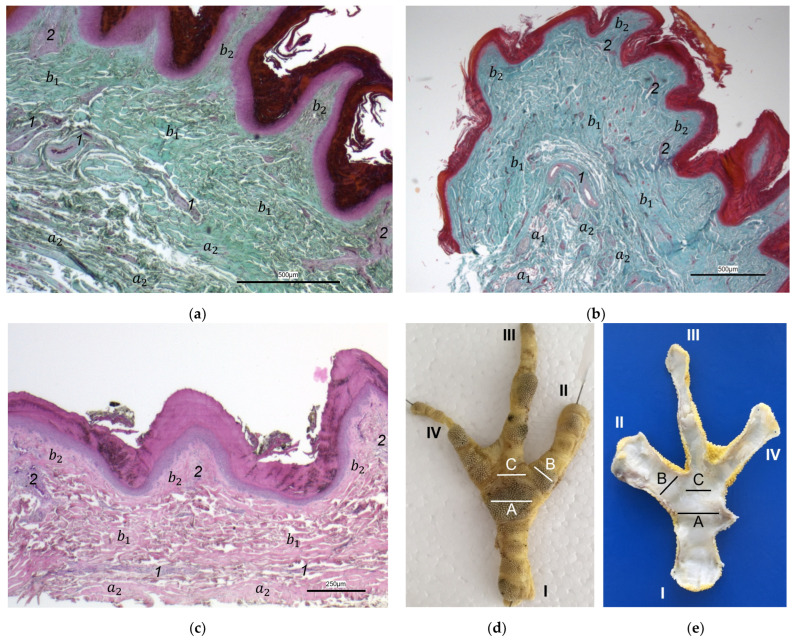
Histological cross-sections of the skin at different localizations of the foot sole in the northern goshawk. Metatarsal foot pad, Masson-Goldner staining (**a**); proximal digital foot pad of the second toe, Masson-Goldner staining (**b**); interpulvinar area cranial to the metatarsal foot pad, HE staining (**c**); right foot, skin of the foot sole, plantar (**d**) and dorsal (**e**) aspect with marked localizations of cross-sections. *a_1_*―fat tissue in the subcutis; *a_2_*―connective tissue in the subcutis; *b_1_*―deep dermal layer; *b_2_*―superficial dermal layer; *1*―blood vessels of subdermal vascular network; *2*―blood vessels of dermal vascular network; A―metatarsal foot pad; B―proximal digital foot pad of the second toe; C―interpulvinar area cranial to the metatarsal foot pad; I–IV―1st to 4th toe.

**Figure 4:**
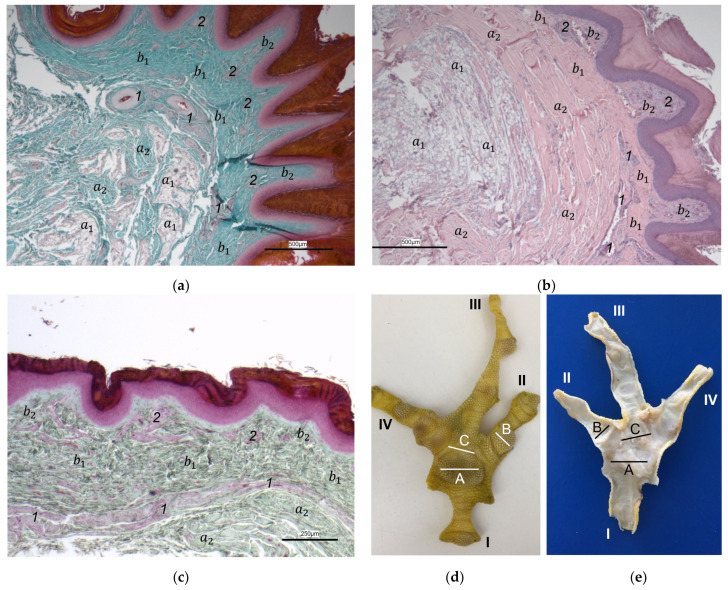
Histological cross-sections of the skin at different localizations of the foot sole in the peregrine falcon. Metatarsal foot pad, Masson-Goldner staining (**a**); proximal digital foot pad of the second toe, HE staining (**b**); interpulvinar area cranial to the metatarsal foot pad, Masson-Goldner staining (**c**); right foot, skin of the foot sole, plantar (**d**) and dorsal (**e**) aspect with marked localizations of cross-sections. *a_1_*―fat tissue in the subcutis; *a_2_*―connective tissue in the subcutis; *b_1_*―deep dermal layer; *b_2_*―superficial dermal layer; *1*―blood vessels of subdermal vascular network; *2*―blood vessels of dermal vascular network; A―metatarsal foot pad; B―proximal digital foot pad of the second toe; C―interpulvinar area cranial to the metatarsal foot pad; I–IV―1st to 4th toe.

**Figure 5:**
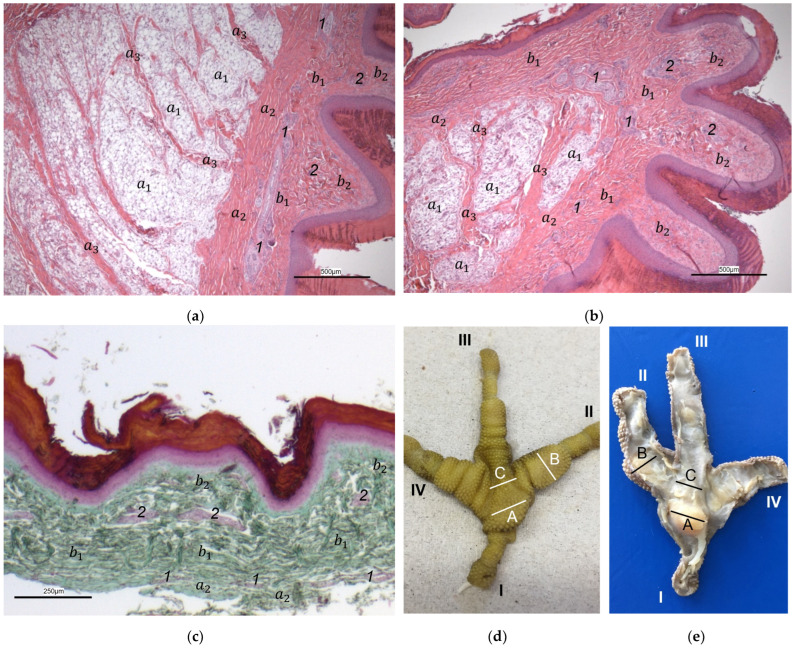
Histological cross-sections of the skin at different localizations of the foot sole in the barn owl. Metatarsal foot pad, HE staining (**a**); proximal digital foot pad of the second toe, HE staining (**b**); interpulvinar area cranial to the metatarsal foot pad, Masson-Goldner staining (**c**); right foot, skin of the foot sole, plantar (**d**) and dorsal (**e**) aspect with marked localizations of cross-sections. *a_1_*―fat tissue in the subcutis; *a_2_*―connective tissue in the subcutis; *a_3_*―connective tissue septa in the fat pad in the subcutis; *b_1_*―deep dermal layer; *b_2_*―superficial dermal layer; *1*―blood vessels of subdermal vascular network; *2*―blood vessels of dermal vascular network; A―metatarsal foot pad; B―proximal digital foot pad of the second toe; C―interpulvinar area cranial to the metatarsal foot pad; I–IV―1st to 4th toe.

The histological examination revealed that the main differences between the species and the localizations examined were found in the **subcutis** (tela subcutanea) with regard to the amount and arrangement of fat tissue (Table 2, Figure 3, Figure 4 and Figure 5).

**Table 2:** Histological examination of the subcutis of the foot sole: number of examined feet classified into categories regarding amount and arrangement of fat tissue at three different localizations (A―metatarsal foot pad, B―proximal digital foot pad of the second toe, and C―interpulvinar area cranial to the metatarsal foot pad) per species.

	Northern Goshawk			Common Buzzard			Peregrine Falcon			Gyr–Saker Falcon			Common Kestrel			Eurasian Eagle-Owl			Long-Eared Owl			Barn Owl		
Amount and arrangement of fat tissue in subcutis	A	B	C	A	B	C	A	B	C	A	B	C	A	B	C	A	B	C	A	B	C	A	B	C
No fat cells in connective tissue (see, e.g., Figure 3a)	10	5	12	10	8	10	0	0	11	1	1	7	0	1	12	0	1	7	0	0	10	0	0	8
Single fat cells or small isolated cluster(s) of accommodated fat cells in connective tissue (see, e.g., Figure 3b)	2	5	0	1	3	0	4	1	1	1	1	4	0	0	0	0	0	2	0	0	1	0	0	2
Prominent organized fat cell cluster(s) in predominant connective tissue (see, e.g., Figure 4a)	0	1	0	0	0	0	7	1	0	5	3	0	0	0	0	0	3	2	1	1	0	0	0	0
Organized fat pad with prominent connective tissue septa (see, e.g., Figure 5a)	0	0	0	0	0	0	1	4	0	3	3	0	12	4	0	12	8	0	11	9	1	12	11	0
Organized fat pad without prominent connective tissue septa (see, e.g., Figure 4b)	0	0	0	0	0	0	0	6	0	2	4	0	0	7	0	0	0	0	0	0	0	0	0	0
Section not evaluable	0	1	0	1	1	2	0	0	0	0	0	1	0	0	0	0	0	1	0	2	0	0	1	2
Total number of feet examined	12			12			12			12			12			12			12			12		

In the majority of examined common buzzards and northern goshawks, the subcutis was classified into the categories “No fat cells” or “Single fat cells or small cluster(s) of fat cells” in the two foot pad regions examined as well as in the interpulvinar area cranial to the metatarsal pad. The subcutis mainly consisted of thick bundles of collagenous fibers with interspersed fibrocytes in these two species (Table 2, Figure 3). In contrast, in the examined falcon species (peregrine falcon, gyr–saker falcon, and common kestrel) and owl species (Eurasian eagle-owl, long-eared owl, and barn owl) organized fat tissue, surrounded by a connective tissue capsule, was found as the basis for the metatarsal pad as well as for the proximal digital pad of the second toe in the majority of the examined specimens. Therefore, the subcutis was mostly classified into the categories “Prominent organized fat cell cluster(s)” or “Organized fat pad”—with or without connective tissue septa (Table 2, Figure 4a,b and Figure 5a,b). Almost all owl specimens showed an organized fat pad that was clearly interspersed with connective tissue septa in both foot pad regions examined. A more heterogeneous picture was observed in the falcon species, e.g., the subcutis of the metatarsal pad was classified as “Prominent organized fat cell cluster(s)” in the largest number of peregrine falcons and gyr–saker falcons, while in the common kestrel, all examined specimens belonged to the category “Organized fat pad with prominent connective tissue septa”. In general, similar to the common buzzard and the northern goshawk, the interpulvinar area cranial to the metatarsal pad showed few or even no fat cells in the majority of all examined falcon and owl species (Table 2, Figure 4c and Figure 5c). Only in two specimens of Eurasian eagle-owls, the subcutis in the interpulvinar area cranial to the metatarsal pad was classified into the category “Prominent organized fat cell cluster(s)” and in one foot of the long-eared owl even into the category “Organized fat pad with prominent connective tissue septa” (Table 2).

In all species examined, the histological examination showed no prominent blood vessels in the subcutis; this observation also included, in particular, the fat pads in falcons and owls.

**Figure 6:**
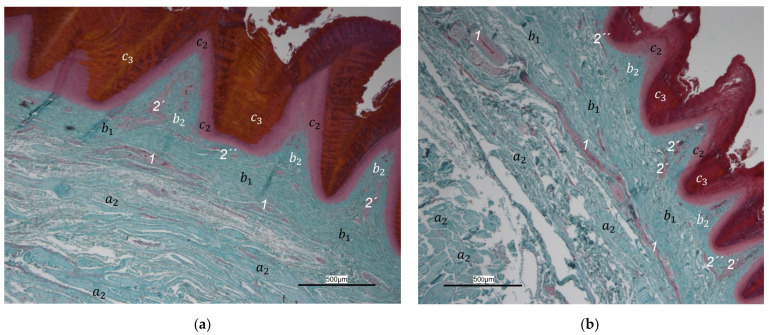

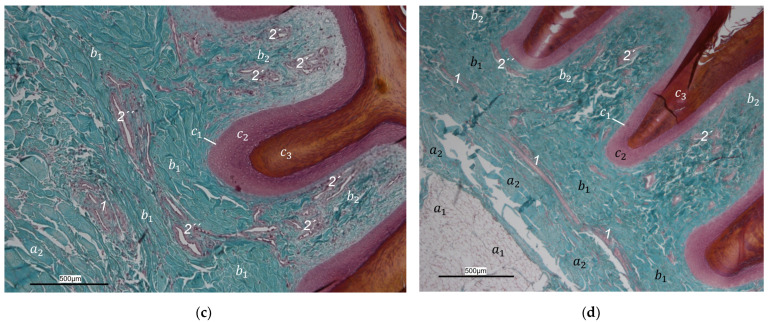
Histological sections of the skin showing the superficial and deep dermal layers and the dermal and subdermal vascular networks. Common buzzard, longitudinal section through the metatarsal foot pad, Masson-Goldner staining (**a**); northern goshawk, cross-section through the proximal digital foot pad of the second toe, Masson-Goldner staining (**b**); peregrine falcon, cross-section through the metatarsal foot pad, Masson-Goldner staining (**c**); Eurasian eagle-owl, longitudinal section through the metatarsal foot pad, Masson-Goldner staining (**d**). *a_1_*―fat tissue in the subcutis; *a_2_*―connective tissue in the subcutis; *b_1_*―deep dermal layer; *b_2_*―superficial dermal layer; *1*―blood vessels of subdermal vascular network; *2’*―blood vessels of dermal vascular network, papillary part; *2’’*―blood vessels of dermal vascular network, interpapillary part; *c_1_*―epidermal basal layer*; c_2_*―epidermal intermediate layer; *c_3_*―epidermal cornified layer.

**Figure 7:**
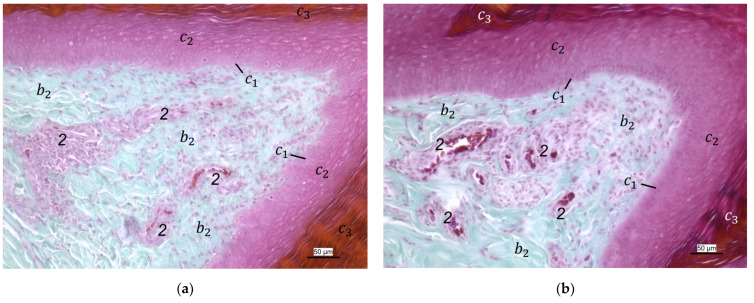

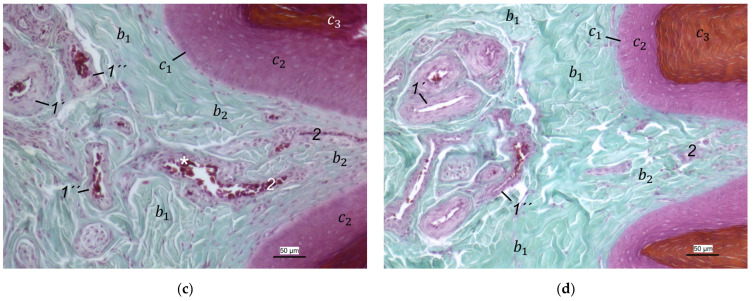
Histological sections of the skin showing the superficial (**a**–**d**) and deep (**c**,**d**) dermal layers and the dermal and subdermal vascular networks, Masson-Goldner staining. Common buzzard, longitudinal section through the metatarsal foot pad (**a**); peregrine falcon, longitudinal section through the proximal digital foot pad of the second toe (**b**), cross-section through the metatarsal foot pad (**c**) and cross-section through the proximal digital foot pad of the second toe (**d**); *b_1_*―deep dermal layer; *b_2_*―superficial dermal layer; *1’*―artery of subdermal vascular network; *1’’*―vein of subdermal vascular network; *2*―blood vessels of dermal vascular network; *―papillary blood vessel emerging from subdermal vascular network into dermal papilla; *c_1_*―epidermal basal layer; *c_2_*―epidermal intermediate layer; *c_3_*―epidermal cornified layer.

**Figure 8:**
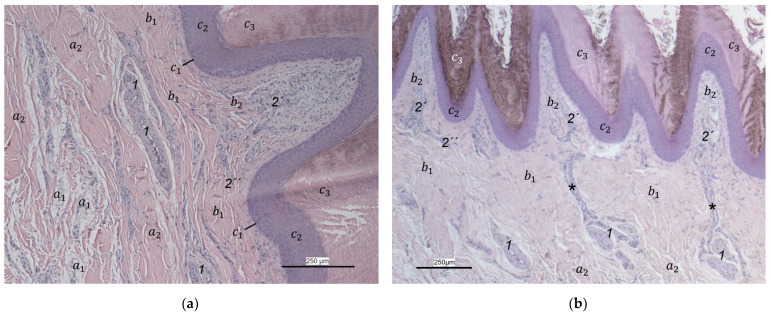

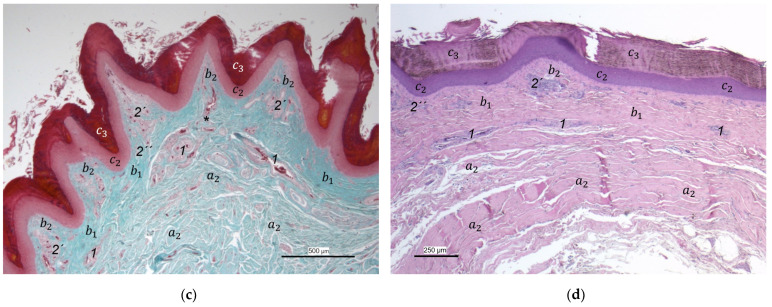
Histological sections of the skin showing the dermal and subdermal vascular networks in the peregrine falcon in the three different localizations examined. Cross-section through the metatarsal foot pad, HE staining (**a**,**b**); longitudinal section through the proximal digital foot pad of the second toe, Masson-Goldner staining (**c**); cross-section through the interpulvinar area cranial to the metatarsal foot pad, HE staining (**d**); *a_1_*―fat tissue in the subcutis; *a_2_*―connective tissue in the subcutis; *b_1_*―deep dermal layer; *b_2_*―superficial dermal layer; *1*―blood vessels of subdermal vascular network; *2’*―blood vessels of dermal vascular network, papillary part; *2’’*―blood vessels of dermal vascular network, interpapillary part; *―papillary blood vessels emerging from subdermal vascular network into dermal papillae; *c_1_*―epidermal basal layer; *c_2_*―epidermal intermediate layer; *c_3_*―epidermal cornified layer.

Between the subcutis and the dermis, arteries and veins were identified in the histological sections forming a deep vascular network of the skin, the **subdermal vascular network** (Figure 6 and Figure 7c,d). This vascular network appeared to be more prominent in the two foot pad regions examined than in the interpulvinar area cranial to the metatarsal pad (Figure 8). Out of this subdermal vascular network, blood vessels emerged into the overlying dermis: the papillary arteries and veins (Figure 7c and Figure 8b,c).

**Figure 9:**
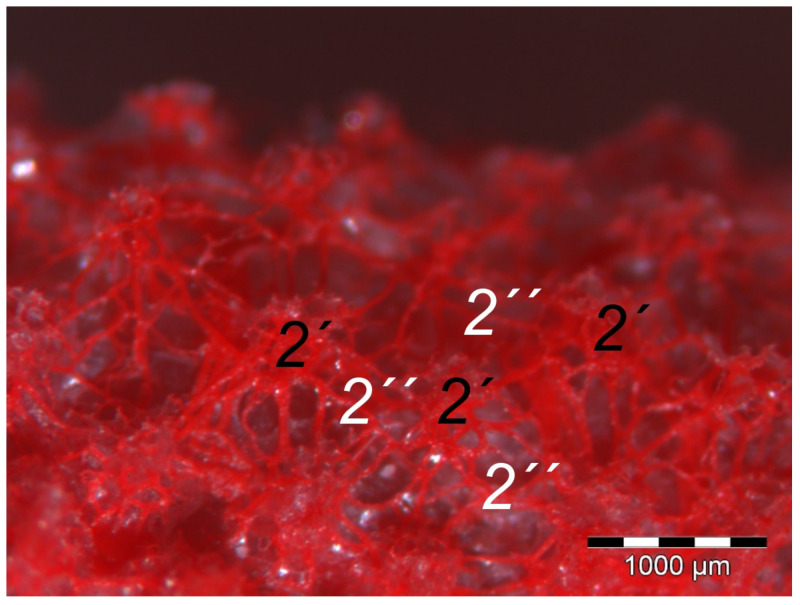
Corrosion cast of the papillary and interpapillary part of the dermal vascular network after maceration, blood vessels filled with epoxy resin. Common buzzard, metatarsal foot pad (blood vessels of papillary part of dermal vascular network visible because subepithelial capillaries not filled with epoxy resin); *2’*―blood vessels of dermal vascular network, papillary part; *2’’*―blood vessels of dermal vascular network, interpapillary part.

The **dermis** histologically consisted of two layers, a deep dermal layer and a superficial dermal layer (Figure 6, Figure 7c,d and Figure 8). On the sole of the foot, the superficial dermal layer formed dermal papillae. The papillary arteries and veins originating from the subdermal vascular network crossed the deep dermal layer to branch out at the base of the papillae and to release dense bundles of arterioles and venules into the center of the papillae. The vascular bundles of each papilla were connected to each other via several interpapillary branches. Thus, this superficial vascular network of the skin, the **dermal vascular network**, consisted of two parts, the papillary and the interpapillary part (Figure 6 and Figure 8). The dermal vascular network was also clearly visible in the corrosion casts (Figure 9).

The appearance of the superficial dermal layer varied between the three localizations examined. The superficial dermal layer of the metatarsal foot pad and the proximal digital foot pad of the second toe showed more prominent papillae than the interpulvinar area cranial to the metatarsal pad. In the interpulvinar area cranial to the metatarsal pad, the papillae seemed to be flatter as well as lower in number and, accordingly, contained less well-developed vascular bundles (Figure 3, Figure 4, Figure 5 and Figure 8).

**Figure 10:**
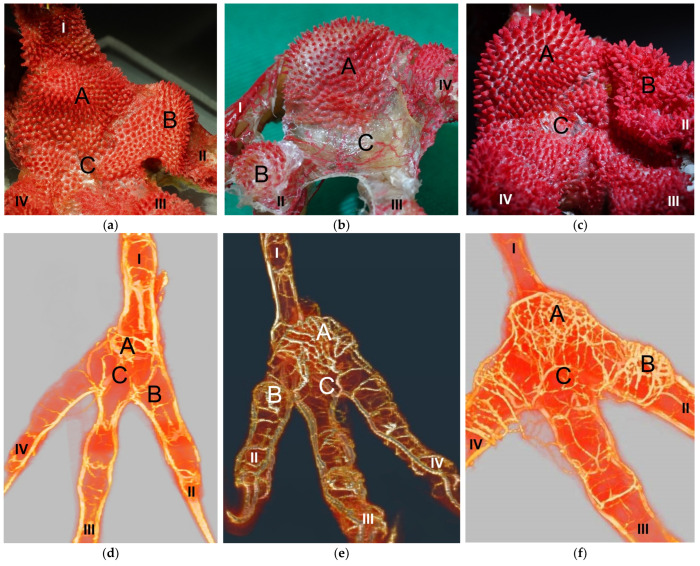
Corrosion casts of the pedal vessels after maceration showing the blood vessels of the dermal papillae in the three localizations examined, blood vessels filled with epoxy resin (**a**–**c**); images of µCT 3D reconstructions showing the blood vessels of the foot including the subdermal vascular networks of the foot pads, arteries and veins filled with contrast medium (**d**–**f**). Common buzzard, left foot, plantar aspect (**a**,**d**); gyr–saker falcon, right foot, plantar aspect (**b**,**e**); Eurasian eagle-owl, left foot, plantar aspect (**c**,**f**); A―metatarsal foot pad; B―proximal digital foot pad of the second toe; C―interpulvinar area cranial to the metatarsal foot pad; I–IV―1st to 4th toe.

In summary, the two foot pads showed more prominent dermal papillae and a more prominent dermal and subdermal vascular supply in comparison to the interpulvinar area cranial to the metatarsal pad (Figure 10). The dermal layers and vascular networks were similar in all species studied. In comparison, the dermal layers showed a more prominent vascular supply than the subcutis.

**Figure 11:**
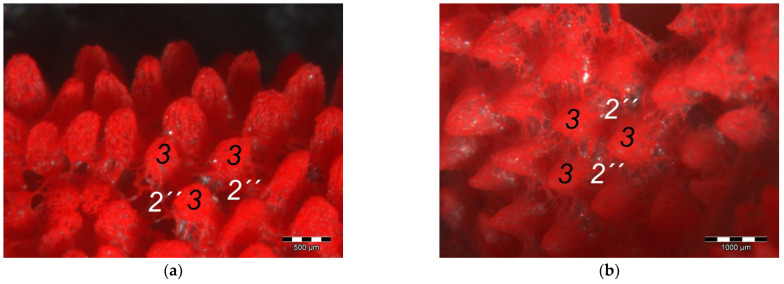
Corrosion casts of the subepithelial layer of fine capillaries covering the dermal papillae after maceration, blood vessels filled with epoxy resin, proximal digital foot pad of the second toe. Long-eared owl (**a**); Eurasian eagle-owl (**b**); *2*’’―blood vessels of dermal vascular network, interpapillary part; *3*―subepithelial layer of capillaries emerging from the papillary part of the dermal vascular network.

**Figure 12:**
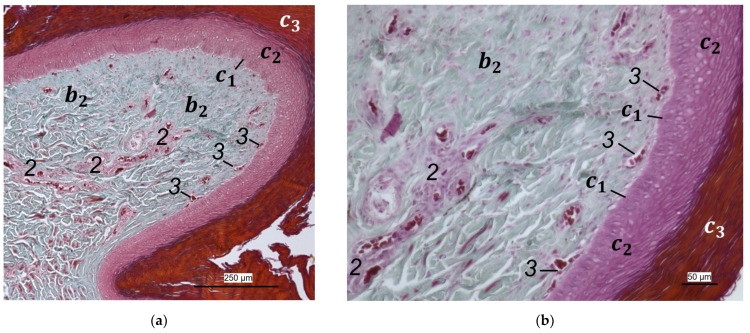

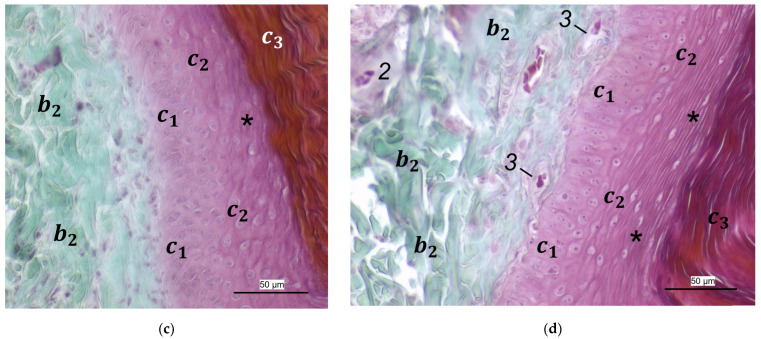
Histological sections of the epidermis, Masson-Goldner staining. Eurasian eagle-owl, longitudinal section through the metatarsal foot pad (**a**,**b**); barn owl, longitudinal section through the proximal digital foot pad of the second toe (**c**); common kestrel, longitudinal section through the area cranial to the metatarsal foot pad (**d**); *b_2_*―superficial dermal layer; *2*―blood vessels of dermal vascular network; *3*―subepithelial capillaries emerging from the papillary part of the dermal vascular network; *c_1_*―epidermal basal layer; *c_2_*―epidermal intermediate layer; *c*_3_―epidermal cornified layer; *―flattening cells of the epidermal intermediate layer.

Out of the papillary part of the dermal vascular network in the center of the dermal papillae, very fine blood vessels emerged that formed a **subepithelial layer of fine capillaries**. The subepithelial capillaries covered the dermal papillae on their entire surface. As a result, the injection material filling the subepithelial capillaries formed exact replicas of the dermal papillae on the surface of the corrosion casts (Figure 11). Sections of these capillaries were visible in the histological specimens in the peripheral area of the dermal papillae directly below the epidermis (Figure 12).

In the **epidermis**, the same three layers were identified at all three localizations examined: a basal, an intermediate, and a cornified layer (Figure 12). The basal layer was deepest and consisted of a single layer of cells. In the intermediate layer, the cells flattened towards the cornified layer. All species examined showed a thick cornified layer, which could be clearly differentiated against the intermediate layer. This arrangement of epidermal layers was the same in all species studied. The epidermal papillae corresponded to the shape of the underlying dermal papillae and reflected their number and size. In consequence, the macroscopically visible epidermal papillae on the foot sole were more prominent in the regions of the foot pads in comparison to the interpulvinar area cranial to the metatarsal pad (Figure 1).

### 3.2. Vasculature of the Dorsal and Plantar Side of the Toes Including Digital Foot Pads

**Figure 13:**
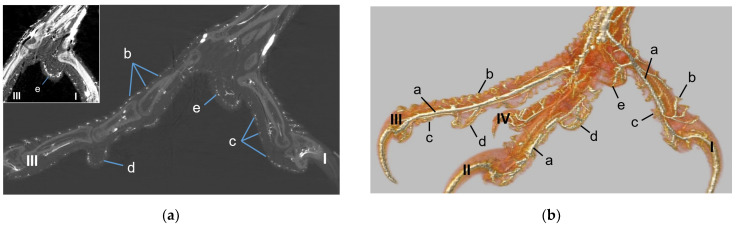

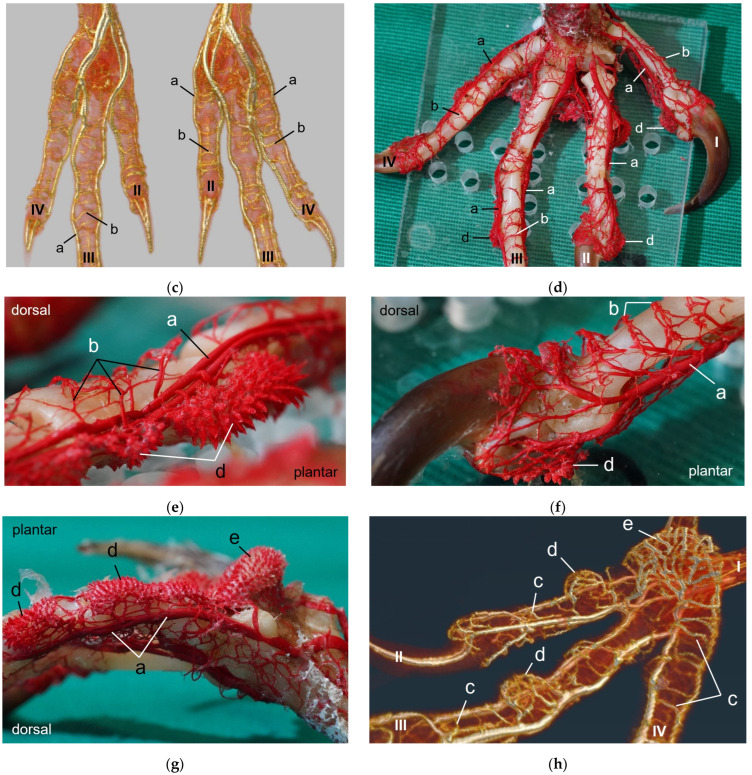
Vasculature of the dorsal and plantar aspect of the toes in peregrine falcon (**a**–**c**) and gyr–saker falcon (**d**–**h**). µCT scans, blood vessels filled with contrast medium (**a**–**c**,**h**); longitudinal section centered through toe I and toe III with the metatarsal foot pad sectioned peripherally (inset: section through the center of the metatarsal foot pad), right foot (**a**); images of 3D reconstructions, right foot, medial aspect (**b**), pair of feet, dorsal aspect (**c**), and right foot, plantar aspect (**h**). Corrosion casts of the pedal vessels after maceration, blood vessels filled with epoxy resin, small vessels of the dorsal skin removed, right foot (**d**–**g**); dorsal aspect (**d**); lateral aspect of the third (**e**), medial aspect of the second (**f**) and lateral aspect of the fourth toe (**g**); a―lateral and medial digital arteries and veins; b―their brace-like branches over the dorsal aspect of the toes; c―their brace-like branches over the plantar aspect of the toes; d―branches of the brace-like branches ramifying in the skin of the plantar digital foot pads as subdermal vascular network; e―branches ramifying in the skin of the plantar metatarsal foot pad as subdermal vascular network; I–IV―1st to 4th toe.

In all species examined, each toe was supplied by one digital artery and one digital vein on its lateral as well as on its medial side (Figure 13, see also Schwehn et al., 2024 [27]). The dorsal and plantar skin of the toes was vascularized by parallel brace-like branches of these collateral digital arteries and veins. At the plantar surface of the foot, these brace-like branches ramified and encircled the digital foot pads as the subdermal vascular network (Figure 13).

### 3.3. Arterial Pulvinar Branches Supplying the Metatarsal Foot Pad

Several digital arteries and the arterial plantar arch released branches for the supply of the metatarsal foot pad, which were referred to as pulvinar arteries or arterial pulvinar branches. Depending on the method of investigation and avian species, the extent to which these blood vessels was filled with latex or contrast medium varied, and thus the number of pulvinar branches visible in each specimen also differed. The number of visible arterial pulvinar branches in relation to the total number of feet examined is given in Table 3. For an easier readability, we used the following abbreviations for the arteries.
co_dMACommon dorsal metatarsal artery;la_dMALateral dorsal metatarsal artery;me_dMAMedial dorsal metatarsal artery;mi_dMAMiddle dorsal metatarsal artery;la_DA1-4Lateral digital artery of the first to fourth toe;me_DA1-4Medial digital artery of the first to fourth toe;pA1-4Arterial pulvinar branches from digital arteries (beginning medial, proceeding to lateral);pA5Arterial pulvinar branch from arterial plantar arch.

**Figure 14:**
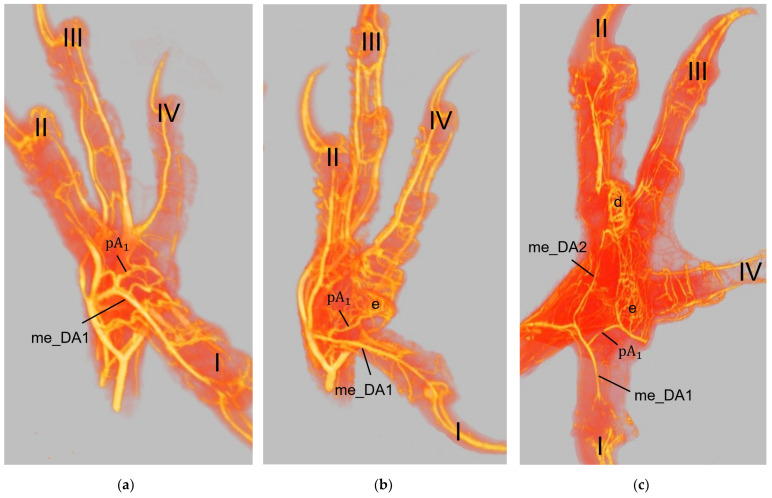

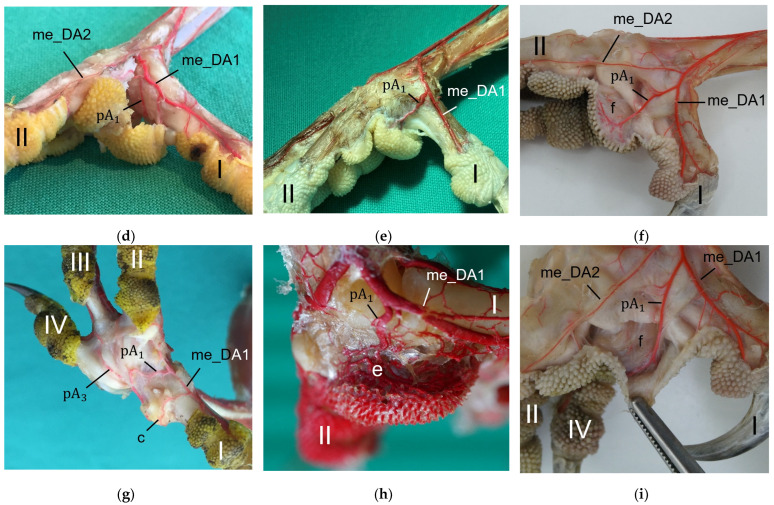
Main arterial pulvinar branch arising from the me_DA1 supplying the metatarsal foot pad. Images of µCT 3D reconstructions, blood vessels filled with contrast medium, left foot, plantomedial aspect in common buzzard (**a**), peregrine falcon (**b**), and Eurasian eagle-owl (**c**). Right foot of common buzzard after removal of skin and extensor tendons, arteries filled with red-colored latex, medial (**d**) and plantar (**g**) aspect; right foot of gyr–saker falcon after removal of skin and extensor tendons, arteries filled with red-colored latex, medial aspect (**e**) and corrosion cast after maceration, blood vessels filled with epoxy resin, medial aspect (**h**); right foot of Eurasian eagle-owl after removal of skin and extensor tendons, arteries filled with red-colored latex, medial aspect (**f**,**i**). me_DA1―medial digital artery of the first toe; me_DA2―medial digital artery of the second toe; pA1―arterial pulvinar branch from the me_DA1; pA3―arterial pulvinar branch from the interdigital space between toes III and IV; c―brace-like branch from the me_DA1 over the plantar aspect of the first toe; d―branches of the brace-like branches ramifying in the skin of the proximal digital foot pad of the second toe (subdermal vascular network); e―branches from the pulvinar branch of the me_DA1 ramifying in the skin of the metatarsal foot pad (subdermal vascular network); f―fat tissue as the basis of the metatarsal foot pad; I–IV―1st to 4th toe.

Arterial pulvinar branches arising from the digital arteries could be allocated to the four interdigital spaces, resulting in three possible origins per interdigital space: the two flanking digital arteries themselves and the splitting point of their original vessel. In the **interdigital space between toes I and II**, the by-far-largest arterial branch for the metatarsal pad was observed to originate from the strong medial digital artery of the first toe (me_DA1) in all species examined (Figure 14). However, the course of this pulvinar branch differed between species: In the examined owls and falcons, it ran in a plantar direction vertically to the sole surface and encircled the fat pad, which was more prominent in these species as mentioned above. In the skin of the metatarsal pad, it ramified into several small branches, forming the subdermal vascular network and surrounding the plantar surface of the fat pad like a basket. This basket-like distribution pattern was clearly visible in the µCT scans, with only a few vessels visible within the basket, where the fat pad was located in falcons and owls. In northern goshawks and common buzzards, the main arterial branch for the metatarsal pad ran almost horizontally to the sole surface in a straight course in the lateral direction; small vessels supplying the skin emerged from this arterial branch. Therefore, its course resembled more the course of the brace-like branches arising from the collateral digital arteries and supplying the skin of the plantar surface of the toes; a basket-like distribution pattern was missing in northern goshawks and common buzzards (Figure 14). While the strong pulvinar branch from the me_DA1 was clearly visible in all examined species and almost all examined feet (n = 71/75), the small, medial digital artery of the second toe (me_DA2) gave off a small pulvinar branch only in five feet of Eurasian eagle-owls and long-eared owls. In three of these cases, the pulvinar branch from the me_DA2 was present in addition to the branch from the me_DA1; in two cases, however, it replaced the pulvinar branch of the me_DA1.

**Figure 15:**
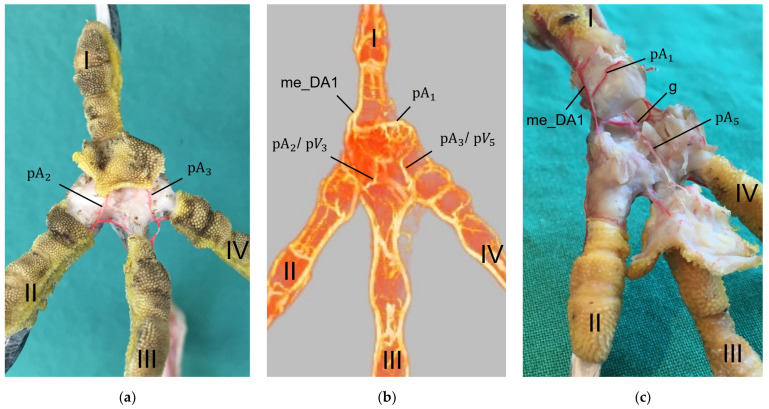

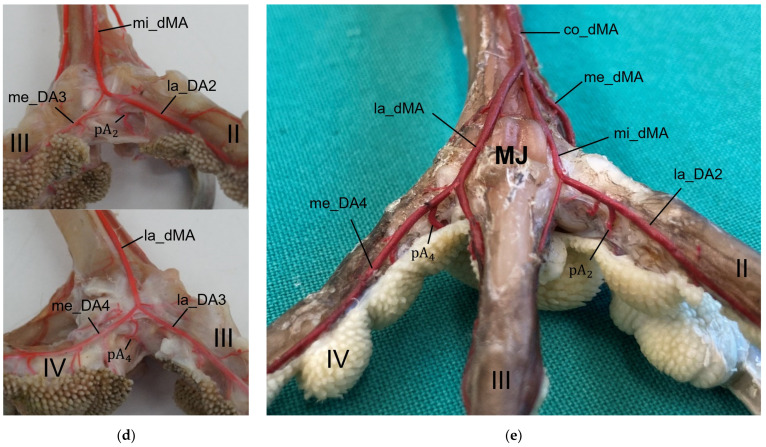
Minor arterial pulvinar branches supplying the metatarsal foot pad. Right foot of northern goshawk after removal of skin, arteries filled with red-colored latex, plantar aspect (**a**); right foot of peregrine falcon, image of µCT 3D reconstruction, blood vessels filled with contrast medium, plantar aspect (**b**); right foot of common buzzard after removal of skin and flexor tendons, arteries filled with red-colored latex, plantar aspect (**c**); right foot of Eurasian eagle-owl (**d**) and gyr–saker falcon (**e**) after removal of skin and extensor tendons, arteries filled with red-colored latex, dorsal aspect. co_dMA―common dorsal metatarsal artery; la_dMA―lateral dorsal metatarsal artery; me_dMA―medial dorsal metatarsal artery; mi_dMA―middle dorsal metatarsal artery; me_DA1―medial digital artery of the first toe; la_DA2―lateral digital artery of the second toe; la_DA3―lateral digital artery of the third toe; me_DA3―medial digital artery of the third toe; me_DA4―medial digital artery of the fourth toe; pA1―arterial pulvinar branch from the me_DA1; g―arterial plantar arch; pA2―arterial pulvinar branch from the la_DA2; pA3―arterial pulvinar branch from the interdigital space between toes III and IV; pA4―arterial pulvinar branch from the med_DA4; pA5―arterial pulvinar branch from the arterial plantar arch; pV3―venous pulvinar branch from the interdigital space between toes II and III; pV5―venous pulvinar branch from the interdigital space between toes III and IV; MJ―metatarsophalangeal joints; I–IV―1st to 4th toe.

Further small arterial pulvinar branches supplying the metatarsal pad were released from arteries in the interdigital spaces between toes II and III as well as between toes III and IV (Figure 15).

In the **interdigital space of toes II and III**, the strong, lateral digital artery of the second toe (la_DA2) and the small, medial digital artery of the third toe (me_DA3) originated from the splitting of the middle dorsal metatarsal artery (mi_dMA). Here, pulvinar branches arose from either the la_DA2, the me_DA3 or the point of their origin. The majority of feet examined (n = 61/75) showed a pulvinar branch arising from the la_DA2. The pulvinar branch from the la_DA2 remained the only one in all examined feet of the common buzzards, northern goshawks, peregrine falcons, and common kestrels as well as in five feet of the Eurasian eagle-owls, six feet of the long-eared owls, and all but one foot of the gyr–saker falcons. Five examined feet of the Eurasian eagle-owls as well as two feet of the long-eared owls showed the pulvinar branch from the la_DA2 and an additional pulvinar branch from the me_DA3. In the gyr–saker falcons, in all specimens, the pulvinar branch from the la_DA2 was visible, and, in one single foot, an additional pulvinar branch emerged from the origin of the la_DA2 and me_DA3. In the barn owl, four examined specimens showed pulvinar branches, both from the la_DA2 and the me_DA3, but, in two specimens, there were only pulvinar branches from the la_DA2 and, in three specimens, only from the me_DA3.

In the **interdigital space of toes III and IV**, the lateral digital artery of the third toe (la_DA3) and the medial digital artery of the fourth toe (me_DA4), both strong arteries, originated from the splitting of the lateral dorsal metatarsal artery (la_dMA). Here—again—pulvinar branches arose from either the la_DA3, the me_DA4, or the point of their origin. In the majority of feet examined (n = 48/75), a pulvinar branch arising from the me_DA4 was present. In barn owls, gyr–saker falcons, and common kestrels, this branch remained the only one. In the peregrine falcon, two feet showed an additional pulvinar branch from the la_DA3. Common buzzards, northern goshawks, Eurasian eagle-owls, and long-eared owls showed additional pulvinar branches, too, either arising from the la_DA3 in seven feet or from the origin of the me_DA4 and la_DA3 in nine feet.

Arterial pulvinar branches from the arteries of the **interdigital space between toes IV and I** were extremely rare. A very small pulvinar branch for the metatarsal pad was visible arising from the slender lateral digital artery of the fourth toe (la_DA4) in one single specimen of the Eurasian eagle-owl.

In addition, the **arterial plantar arch** gave rise to rather small pulvinar branches. However, this was only the case in a few specimens of different species (10/75) and did not occur noticeably more often in species that had a strong plantar arch, which are the common buzzard, the northern goshawk, and the barn owl (Figure 15). This arterial plantar arch arose from the distal intermetatarsal artery passing through the distal vascular foramen of the tarsometatarsus. The arterial plantar arch was located at the level of the metatarsophalangeal joints and underneath the flexor tendons, profound to the metatarsal pad (Figure 15, see also Schwehn et al., 2024 [27]). In this region, almost no vessels were visible around the flexor tendons (Figure 13a).

### 3.4. Venous Pulvinar Branches Draining the Metatarsal Foot Pad

Several digital veins as well as the venous plantar arch received venous branches from the metatarsal foot pad, which were referred to as pulvinar veins or venous pulvinar branches. Corresponding to the description of the arteries, Table 4 shows the number of visible venous pulvinar branches in relation to the total number of feet examined. For an easier readability, we used the following abbreviations for the veins.
me_pMVMedial plantar metatarsal vein;la_dCDVLateral dorsal common digital vein;me_dCDVMedial dorsal common digital vein;la_pCDVLateral plantar common digital vein;me_pCDVMedial plantar common digital vein;la_DV1-4Lateral digital vein of the first to fourth toe;me_DV1-4Medial digital vein of the first to fourth toe;pV1-7Venous pulvinar branches from digital veins (beginning medial, proceeding to lateral).

**Figure 16:**
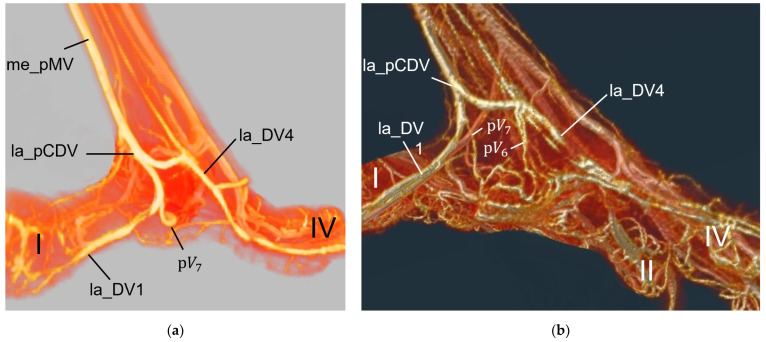

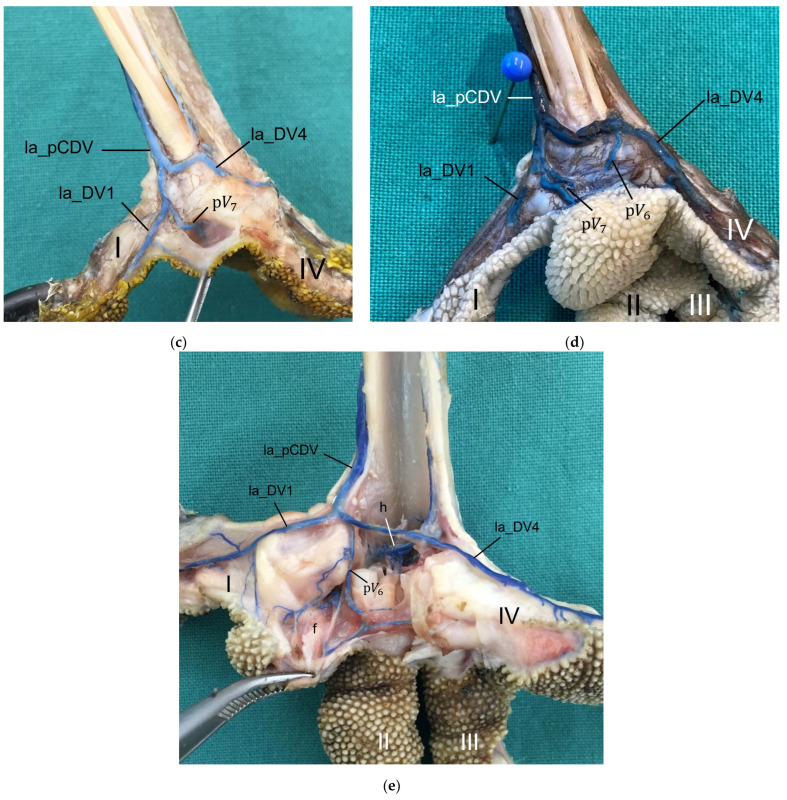
Main venous pulvinar branch(es) joining the la_DV1 or the la_DV4 and draining the metatarsal foot pad. Lateral aspect of the right foot in common buzzard (**a**) and gyr–saker falcon (**b**), images of µCT 3D reconstructions, blood vessels filled with contrast medium; lateral aspect of the right foot in common buzzard (**c**) and gyr–saker falcon (**d**) after removal of skin, veins filled with blue-colored latex; lateral aspect of the right foot in Eurasian eagle-owl after removal of skin and flexor tendons, first toe displaced medially, veins filled with blue-colored latex (**e**); me_pMV―medial plantar metatarsal vein; la_pCDV―lateral plantar common digital vein; la_DV1―lateral digital vein of the first toe; la_DV4―lateral digital vein of the fourth toe; h―venous plantar arch; pV6―venous pulvinar branch joining the la_DV4; pV7―venous pulvinar branch joining the la_DV1; f―fat tissue as the basis of the metatarsal foot pad; I–IV―1st to 4th toe.

Similar to the pulvinar arteries, in the four interdigital spaces, the venous drainage of the metatarsal pad was via venous pulvinar branches joining into the digital veins, resulting in three possible connection points per interdigital space: the two flanking digital veins themselves and the point of their junction. The main drainage of the metatarsal foot pad was found in the **interdigital space between toes I and IV** and took place via a branch of the lateral digital vein of the first toe (la_DV1) or the lateral digital vein of the fourth toe (la_DV4), both being strong digital veins. As a result, the main venous drainage was located laterally, opposite to the medially positioned main arterial supply via the me_DA1 (Figure 16). The main venous drainage differed between species and even individually between the specimens examined. The la_DV4 ran proximally along the fourth toe to the level of the metatarsophalangeal joints. There, it curved like an arch superficially around the flexor tendons to join the la_DV1. The pulvinar branch joined the curved part of the la_DV4 (n = 34/75) or the la_DV1 near its junction with the la_DV4 (n = 20/75). In all examined specimens of barn owls, common buzzards, and common kestrels, in which the vessel was visible, the branch joining into the la_DV1 remained the only one. In Eurasian eagle-owls and long-eared owls, the drainage was found to be via the la_DV1 in four feet and via the la_DV4 in ten feet. Northern goshawks, peregrine falcons and gyr–saker falcons showed more variations. In the northern goshawk, there was a pulvinar branch draining into the la_DV1 in eight feet, with an additional branch draining into the la_DV4 in two cases, while in one foot, there was only a pulvinar branch draining into the la_DV4. In the peregrine falcon, there was a pulvinar branch joining the la_DV1 in five feet, with an additional pulvinar branch joining the la_DV4 in three cases, while in one foot, there was only a pulvinar branch joining the la_DV4. In the gyr–saker falcon, drainage was via a pulvinar branch that joined the la_DV1 in four feet, showing an additional branch joining the la_DV4 in two cases, while in one foot, the drainage was only via a pulvinar branch that joined the la_DV4.

**Figure 17:**
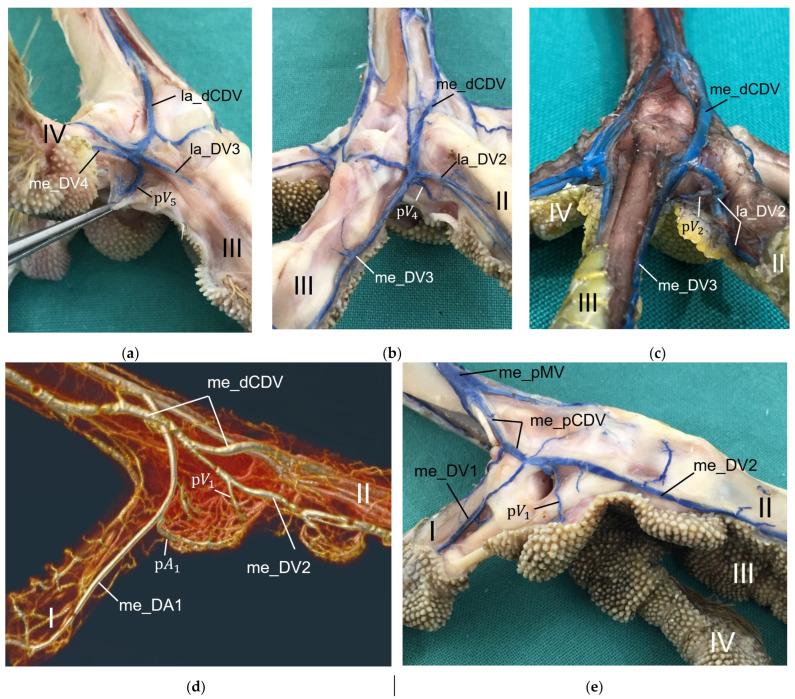
Minor venous pulvinar branches draining the metatarsal foot pad. Dorsal aspect of the right foot in Eurasian eagle-owl (**a**,**b**) and peregrine falcon (**c**) after removal of skin and partly removal of extensor tendons, veins filled with blue-colored latex. Left foot of gyr–saker falcon, µCT 3D reconstruction, blood vessels filled with contrast medium, medial aspect (**d**). Left foot of Eurasian eagle-owl after removal of skin, veins filled with blue-colored latex, medial aspect (**e**). me_pMV―medial plantar metatarsal vein; la_dCDV―lateral dorsal common digital vein; me_dCDV―medial dorsal common digital vein; me_pCDV―medial plantar common digital vein; me_DA1―medial digital artery of the first toe; me_DV1―medial digital vein of the first toe; la_DV2―lateral digital vein of the second toe; me_DV2―medial digital vein of the second toe; la_DV3―lateral digital vein of the third toe; me_DV3―medial digital vein of the third toe; me_DV4―medial digital vein of the fourth toe; pA1―arterial pulvinar branch from me_DA1; pV1―venous pulvinar branch from med_DV2; pV2―venous pulvinar branch from la_DV2; pV4―venous pulvinar branch from med_DV3; pV5―venous pulvinar branch from interdigital space between toes III and IV; I–IV―1st to 4th toe.

Similar to the arterial pattern, additional minor venous pulvinar branches draining the metatarsal pad joined the digital veins in the interdigital spaces between toes IV and III as well as between toes III and II (Figure 17).

In the **interdigital space of toes IV and III**, the medial digital vein of the fourth toe (me_DV4) and the lateral digital vein of the third toe (la_DV3), both small veins, confluenced and continued proximally as lateral dorsal common digital vein (la_dCDV). Here, the pulvinar branch joined mainly the me_DV4 (n = 8/75) or less often the la_DV3 (n = 2/75), or the point of their junction (n = 4/75). In the Eurasian eagle-owl, the pulvinar branch drained into the me_DV4 in four feet, with an additional branch draining into the la_DV3 in one case, and, in two feet, the pulvinar branch drained into the junction of the la_DV4 and la_DV3. In the common kestrel, none of the examined specimens showed pulvinar branches joining the digital veins in the interdigital space between toes IV and III.

In the **interdigital space of toes III and II**, the strong, medial digital vein of the third toe (me_DV3) and the small, lateral digital vein of the second toe (la_DV2) confluenced and continued proximally as medial dorsal common digital vein (me_dCDV). In the majority of feet, the pulvinar branch joined the me_DV3 (n = 12/75) or less frequently the la_DV2 (n = 6/75), or the point of their junction (n = 1/75). In the long-eared owl and the common kestrel, none of the examined specimens showed pulvinar branches joining the digital veins in the interdigital space between toes III and II.

Venous pulvinar branches draining into the veins of the **interdigital space between toes II and I** were weakly developed; in all specimens of the barn owl and of the peregrine falcon, they were even completely absent. When present, in some individuals of the other species examined, a very slender branch from the metatarsal pad was visible to join the strong, medial digital vein of the second toe (me_DV2). This was only the case in 13 of 75 feet examined, seven of which were from common buzzards or northern goshawks. However, in three feet of the common buzzard and one foot of the northern goshawk, the branch joined the small, medial digital vein of the first toe (me_DV1) instead. Taken together, this meant a higher number of venous pulvinar branches, though rather small, on the medial side of the foot in these two species (common buzzard, northern goshawk) compared to the other species studied (Figure 17).

In addition, a small pulvinar branch from the metatarsal pad merged with the **venous plantar arch** in four feet of the Eurasian eagle-owl, one foot of the peregrine falcon, and two feet of the gyr–saker falcon. The venous plantar arch ran collaterally to the arterial plantar arch and thus was located at the level of the metatarsophalangeal joints and underneath the flexor tendons, profound to the metatarsal pad (Figure 16, see also Schwehn et al., 2024 [27]). In this region, hardly any vessels were visible around the flexor tendons (Figure 13a).

## 4. Discussion

Pododermatitis is a common disease in captive birds of prey, especially in falcons kept in falconry [1,2,3,4,5,14]. The disease is characterized by the devitalization of the skin on the plantar aspect of the foot [3,10,11], which seems to be etiologically related to circulatory disorders in the feet [2,3,5]. The raptorial species examined in our study were selected for investigation because they can be taxonomically classified to the three orders of interest, which show differences in their prevalence of pododermatitis [1,3,14,19,20]: Falconiformes—peregrine falcon, gyr–saker falcon, and common kestrel; Accipitriformes—northern goshawk and common buzzard; Strigiformes—Eurasian eagle–owl, long-eared owl, and barn owl [30].

Our study focused on the metatarsal foot pad (pulvinus metatarsalis), which is located on the plantar aspect of the foot plantar to the metatarsophalangeal joints at the distal end of the tarsometatarsus, while the digital foot pads (pulvini digitales) are located on the plantar aspect of the toes plantar to the phalangeal joints [31,32,33,34]. The areas in between the foot pads are referred to as “areae interpulvinares” [32,33,34]. Our investigations showed that in the examined falcon and owl species, the metatarsal and digital pads were protruding and could be clearly distinguished from the interpulvinar areas, while common buzzards and northern goshawks showed a rather even and flat surface of the foot sole with less protrusive foot pad regions. In the longitudinal sections through the feet, we found no macroscopic differences in the epidermis and dermis between the species examined. However, the subcutis of the metatarsal pad varied between species in its characteristic, leading to differences in the extent of pad protrusion. To determine whether this was due to differences in tissue structure, we performed histological examinations of the skin of the foot sole at three selected localizations: the metatarsal foot pad as well as the interpulvinar area cranial to the metatarsal pad was chosen as pododermatitis often occurs in these localizations (see figures in [2,3,14]). To compare the structure of different foot pads with each other, the proximal digital foot pad of the second toe was also chosen for histological investigation.

In most textbooks of avian histology, the cutis is defined to be divided into the epidermis and dermis; anatomically, the subcutis is a separate layer and located underneath the cutis [31,33,35,36,37]. For simplification purposes, we used the term “skin” to mean the epidermis, dermis, and subcutis together, as previously done by other authors [35,36].

For the two superficial layers of the skin, epidermis, and dermis, the histological examination confirmed the macroscopic impression: Layers and tissue structure were the same in all species examined. We found the epidermis to be an epithelium in which we differentiated three layers. The outer cornified layer (stratum corneum) was prominent and clearly differentiated against the underlying non-cornified epidermal layer; this observation was in accordance with other authors [33,36,37]. We divided the non-cornified layer into a basal (stratum basale) and an intermediate layer (stratum intermedium); the same nomenclature was used by former authors [33,36,37]. The basal layer consisted of a single layer of cuboid cells at the border to the dermis. Within the intermediate layer, a flattening of the cells towards the cornified layer was well visible in all species examined. These observations agree with the description of Lucas and Stettenheim [36], who summarized the two layers of non-cornified, living cells as germinative layer (stratum germinativum). Some authors described an additional transitional layer [33,36]. This stratum transitivum was characterized by the presence of vacuolated cells but could not be clearly equated with the granular layer (stratum granulosum) or glassy layer (stratum lucidum) described in mammals and has, therefore, been given its own term [36]. Other authors, such as Sawyer et al. [38], also saw vacuolated cells but included them in the intermediate layer and did not describe them as a separate layer. In the present study, the formation of vacuoles was not clearly visible, and certainly no separate layer could be identified.

In the dermis, two layers could be distinguished in all species examined in the present study: a superficial and a deep dermal layer. These findings are consistent with previous studies on the avian dermis in which the superficial dermal layer was referred to as stratum superficiale and the deep dermal layer as stratum profundum [33,36,37]. Underneath the epidermal papillae of the foot pads studied, the superficial dermal layer was very well developed and formed prominent dermal papillae. These papillae were present in all species of birds of prey and owls examined and interlocked dermis and epidermis in the regions of the metatarsal pad and proximal digital pad of the second toe. Therefore, we consider it appropriate to use the nomenclature “papillary layer” (stratum papillare) for the stratum superficiale in the regions of the foot pads. According to several authors, the feathered skin in birds lacks papillae as the eponymous structure of a papillary layer (stratum papillare), which is why most authors agree that this nomenclature used in mammals is not appropriate for birds [36,37,38]. Our findings are in accordance with studies stating that in the avian skin, a papillary layer is present only in specific locations, e.g., the foot pads [33,36,39]. Vollmerhaus and Hegner [40] explained this in the domestic chicken by pointing out the correlation of a well-developed papillary layer and the fact that the skin of the foot pads has to withstand an increased mechanical stress. In accordance with Vollmerhaus and Hegner [40], we found the superficial dermal layer was less developed, which means the papillae seemed to be much flatter and lower in number in the area cranial to the metatarsal pad.

The macroscopically visible surface structure of the skin of the foot sole reflected the shape of the superficial dermal layer and, thus, was covered by small epidermal papillae. In the literature, different terms were used describing the grainy looking surface structure covering the skin of the foot sole: The surfaces structure was described as papillae-like [40,41,42,43,44,45], granular [40,41], or reticulate [14,36,37,46]. Similar to the dermal papillae, epidermal papillae were prominent and well visible covering the foot pads, while in the interpulvinar areas between them, the macroscopically visible skin surface structure was flatter. According to the literature, this is necessary to ensure the flexibility of the toes [41,47,48].

In contrast to the epidermis and dermis, the study revealed clear differences in the structure of the subcutis of the skin of the foot sole: The amount and arrangement of fat tissue differed between the species examined and between the three localizations examined. In the studied owl and falcon species, organized fat tissue was the basis for the metatarsal pad and the proximal digital pad of the second toe. This is in accordance with earlier studies on the subcutis in the domestic chicken [39,46] as well as in multi-species studies [37,48], which showed that organized fat tissue forms the basis of the metatarsal and digital pads as “fat bodies”. These are located in the subcutis [33,35,36,37]. The metatarsal pad has been described in detail in the domestic chicken: It consists of three compartments of fat tissue, which are separated by septa of collagenous fibers [40]. In the present study, septa were also seen in the species with an organized fat pad in the metatarsal pad as well as in the digital pads. It is noticeable that septa occurred more often in the examined owl species and the common kestrel than in the peregrine falcon and the gyr–saker falcon. A clear difference to previous studies is that “fat bodies” were missing in some species: The common buzzard and the northern goshawk showed collagenous fiber bundles and only few or even no fat cells at all as the basis for the metatarsal pad. This is in contrast to all previous studies, as no literature was found describing connective tissue instead of fat tissue as the basis of the metatarsal pad in any bird species including birds of prey and owls. Due to our results on the species-specific differences in the presence of organized fat tissue occupying space in the subcutis, we are now able to explain the protrusion of the metatarsal foot pad examined in Falconiformes and Strigiformes in contrast to the rather flat sole surface in the Accipitriformes, which did not show a prominent fat tissue body as the basis for the metatarsal pad. For further research, serial sections of the skin of the foot sole would be particularly interesting in order to describe the distribution of fat and connective tissue in more detail in birds of prey and owls, as previously done in the chicken [40].

Former authors found that the foot pads in birds are located exactly at the most mechanically stressed areas of the foot sole [31,33,40]. The metatarsal pad is fully loaded during walking and perching as described in the domestic chicken [31,33,40]. Therefore, the main function of the metatarsal fat pad is to provide a protective cushion and distribute pressure load [40,46] and, in this way, protect vulnerable subcutaneous structures such as tendons, digital joints, and bones from pressure and injuries [14]. The question arises how and why hawks and buzzards do not rely on the protective function of the metatarsal fat pad as they do not show a prominent fat tissue body in this region. Our hypothesis is that, in the Accipitriformes studied, the mechanical load on the sole of the foot is different from that in falcons and owls. The pressure might be less concentrated on the metatarsal pad and more distributed over the toes due to the formation of a foot arch in the region of the metatarsophalangeal joints. This arch could be shaped by a suspensory apparatus of tendons and ligaments as well as the alignment of the tarsometatarsophalangeal joints and is an interesting topic for future research. In this case, the Accipitriformes examined would not need a prominent fat pad to protect themselves against pressure necrosis in the region of the metatarsal pad. If, in contrast, falcons and owls, which have a prominent metatarsal fat pad, were to sit on a flat surface with highly extended metatarsophalangeal joints not shaping a foot arch in the region of the metatarsophalangeal joints, the load on the metatarsal pad would be higher. This could result in a greater importance of the protective function of the metatarsal fat pad but also increase the risk for pressure overload in this region in falcons and owls, especially in the case of abnormal physical behavior in captivity. This hypothesis should be investigated in further studies by determining the pressure at different points on the sole of the foot during perching and comparing it between different avian species.

In contrast to the foot pad regions examined, the interpulvinar area cranial to the metatarsal pad showed no fat cells in most of the examined species and specimens. These results fit in with Vollmerhaus and Hegner [40], who stated that the interpulvinar areas are less exposed to mechanical stress and, therefore, not protected by pads. It can also be discussed that full-length digital pads along the flexor side of the toes would be uncomfortable as they would impair the mobility and the bending of the joints [41].

In addition to pressure compensation, in the literature, another function is described for the digital pads: They adapt precisely to the underlying ground when the toes are flexed to give the bird a better grip when perching [14,31,33,42]. Lennerstedt [43,44,45,47] stated that the surface of the skin of the foot sole shows species-specific differences in the shape and size of the toe pads: Raptorial species such as goshawks, falcons, and owls have relatively raised toe pads, while parrots or representatives of the order Passeriformes show more flat toe pads. The “protrusional” outstanding toe pads enable actively hunting raptorial species to grasp through layers of hair or feathers and hold prey, which is hard to catch [41,49]. In contrast, as described above, we found clear differences in the extent of the protrusion of the digital foot pads within the raptorial species: The toe pads were much more protrusive in the examined falcons and owls than in the northern goshawk and the common buzzard. We found that in falcons and owls, more fat tissue was present as the basis for the proximal digital pad of the second toe than in the examined goshawks and buzzards. Former studies showed fat tissue as the basis for the toe pads in various species including birds of prey and owls [42,48]. In contrast, the prominent toe pads in the sparrowhawk (*Accipiter nisus*) have been described, consisting of cones of connective tissue instead of fat tissue with the function of holding prey and preventing it from escaping [41]. A lack of fat tissue might indicate that the function of the toe pads is not primarily to distribute pressure. Unfortunately, we only investigated histologically the proximal digital pad of the second toe, which in particular has to be discussed with regard to its function in pressure relief, because it is very close to the metatarsal pad and thus the area of greatest pressure load. For example, in eagles, the proximal digital pad of the second toe is often affected by pressure-induced necrosis [3]. Thus, toe pads on the other toes, as well as in other raptorial species, should be examined histologically to differentiate their role in pressure distribution from their function as a prey-catching tool.

In addition to the skin layers, the focus of this study was the blood vessel supply of the foot sole as the development of bumblefoot appears to be related to circulatory disorders of the feet [2,3,5], as described in the introduction. In a former study on eight species of birds of prey and owls, we already described in detail that each toe was supplied by one digital artery and one digital vein on its lateral as well as on its medial side [27]. This is in accordance to descriptions of several avian species including some species of birds of prey and owls [14,21,50]. In the present study, we were able to show that these digital arteries and veins gave rise to various dorsal and plantar brace-like branches on each side of each toe overspinning the dorsal and plantar side of the toes, which is fully in accordance with a study on the domestic chicken [40]. The blood vessels of the skin originated from these brace-like branches. Older studies mention a particularly prominent vasculature of the dermis [51,52]; Vollmerhaus and Hegner [40] showed different blood vessel networks within the skin in the domestic chicken. Similar to the chicken [40], we found that the plantar brace-like branches split up on the plantar side of the toes forming a vascular network between dermis and subcutis. This network was particularly well developed in the regions of the foot pads at the plantar aspect of the foot. In the chicken, it had the mesh size of the base of the papillae in the papillary layer. This corresponds to the results of our study and is particularly clearly visible in the corrosion casts in all species examined. Vollmerhaus and Hegner [40] referred to this vascular network between dermis and subcutis as “cutaneous vascular network”. We would like to introduce a nomenclature better reflecting the stratigraphic localization of the blood vessels and, therefore, suggest the term “subdermal vascular network”.

We found arteries and veins emerging from this subdermal vascular network and running into the dermis up till the dermal papillae. This fits in with former studies on the domestic chicken, which referred to these vessels as papillary arteries and veins [40]. We found this nomenclature was appropriate and, therefore, adopted it. The branching of the papillary arteries and veins into a superficial vascular network filling the dermal papillae and forming interpapillary connecting branches is also consistent with the domestic chicken [40]. This vascular network was labeled as “subpapillary network” [40]. As this network is not only subpapillary, i.e., beneath the papillae, but also in the center of each papilla, we would like to introduce the term “dermal vascular network”. This nomenclature describes the position of the blood vessels within the dermis. To stress the different appearance, we subdivided a papillary part consisting of dense vascular bundles in the center of the papillae and an interpapillary part connecting those bundles. The organized vascular bundles in all single dermal papillae were also shown in the domestic chicken [39,40,53]. These vascular bundles have been described as glomerular shaped in pigeons [52]. In all raptorial species examined in the present study, subepithelial capillaries emerging out of the papillary part of the dermal vascular network were found. These capillaries were also observed in a former study on the domestic chicken [40]. In summary, the present study confirmed the existence of two vascular networks, one subdermal and one dermal vascular network, as well as a layer of subepithelial capillaries in the regions of the metatarsal pad and the proximal digital pad of the second toe in all examined species of birds of prey and owls. We found no differences in the presence of the vascular networks between the examined species.

Our results fit in with previous descriptions of the domestic chicken, which stated that especially the dermal layer of the plantar pads is characterized by a great abundance of blood vessels [39,40,53]. In contrast, the interpulvinar area cranial to the metatarsal pad exhibited not only less-developed dermal papillae but also less-prominent dermal vascular networks in all avian species examined in the present study. It could be concluded that the blood vessel supply and thus the blood circulation of the skin might be better in the regions of the foot pads than in the interpulvinar areas. A better blood supply, especially through the subepithelial capillaries, would cause a better nutrition and thus faster proliferation and cornification of epidermal cells in the foot pads in contrast to the areas between the foot pads [39]. According to former authors, a stronger cornification is required covering the foot pads as they are the parts of the sole that come into contact with the ground and are, therefore, exposed to higher mechanical stress [36,41,46].

The metatarsal foot pad was supplied by several arterial pulvinar branches, which is in line with descriptions of the domestic chicken [40]. But, in contrast to the chicken, we found that one arterial pulvinar branch (from the me_DA1) was the strongest in all species examined. We showed that the course of this main arterial pulvinar branch differed clearly between species depending on the presence of organized fat tissue in the subcutis of the metatarsal pad. The pulvinar branch took a longer way in the plantar direction vertically to the sole surface and encircled the more elevated metatarsal fat pad basket-like in peregrine falcons, gyr–saker falcons, common kestrels, Eurasian-eagle owls, long-eared owls, and barn owls. In contrast, the course of the pulvinar branch was rather straight in the lateral direction and almost horizontally to the sole surface in the region of the metatarsal pad in the northern goshawk and the common buzzard. In the majority of studies on the blood vessel supply of the foot in birds of prey and owls, arteries of the foot sole including the metatarsal pad were not mentioned at all [14,21,22,23,24,25]. Thus, it remains unclear whether these differences in the course of the main arterial pulvinar branch result in significant species-specific differences in the blood supply to the skin of the metatarsal pad. Due to the shorter and less-ramified course, the blood flow to the skin of the metatarsal pad through the pulvinar artery might be better in the northern goshawk and the common buzzard in contrast to the examined falcons and owls.

In all species examined the main arterial supply of the metatarsal pad was via a prominent pulvinar branch from the strong me_DA1. A picture of the southern caracara (*Caracara plancus*), which belongs to the order Falconiformes, from Oliveira et al. [26] shows an arterial branch on the medial side of the foot running towards the metatarsal pad. Oliveira et al. [26] named it “plantar region artery” but did not examine it further. Based on its course, we assume that it corresponds to the main arterial pulvinar branch we described, which arose from the me_DA1. This would mean that similar to the eight species we investigated, this vessel is also very prominent in the southern caracara. Other publications on the blood vessel supply of the feet in birds of prey and owls [14,21,22,23,24,25] do not describe the origin of arteries supplying the metatarsal pad. In the domestic chicken, the me_DA1 was also described as the origin of an arterial pulvinar branch, but it provided only a small, not a major supply [40]. In the domestic chicken [40,54], both the la_DA3 and the me_DA4 were origins giving off prominent arterial pulvinar branches to supply the metatarsal pad. In waterfowl species, a single strong arterial pulvinar branch originated either from the la_DA3 [55] or from a common trunk of the la_DA3 and me_DA4 [56,57]. Only for the domestic chicken, a strong arterial branch ramifying from the common trunk of the la_DA2 and me_DA3 was described as a further third prominent pulvinar branch [40]. Branches to the metatarsal pad arising from the arteries in the interdigital space between toes II and III as well as from the interdigital space between toes III and IV were also observed in the eight species examined in the present study; however, they were all rather small. Small pulvinar arteries were missing in former descriptions of the pulvinar vasculature of the avian feet [26,55,56,57,58,59], probably because the authors did not go into further detail. Only Vollmerhaus and Hegner [40] showed a small arterial pulvinar branch, as mentioned above. Based on our own findings and those of the other authors mentioned, we can state that the main arterial branches for the metatarsal pad always originated from strong digital arteries, both in the birds of prey and owls studied, as well as in domestic chicken [40] and waterfowl [55,56,57]. Furthermore, it is noticeable that the origins of the pulvinar arteries were located mainly in the interdigital spaces between toes I and II, toes II and III, and toes III and IV, both in the eight raptorial species examined, as well as in domestic chicken [40] and waterfowl [55,56,57]. In summary, the distribution pattern of the arterial branches supplying the metatarsal pad varied between species—and even individuals.

In a former study, we investigated the origin of the digital arteries in the same eight avian species as in the present study [27]. We found that four digital arteries (la_DA2, me_DA3, la_DA3, and me_DA4) mentioned as origins for the pulvinar arterial branches, originated from the dorsal metatarsal arteries in all eight species examined. However, the origin of the me_DA1 differed between species. We found that the me_DA1 was a direct continuation of one of the strong dorsal metatarsal arteries in the Eurasian eagle-owl, the long-eared owl, and all three examined falcon species. This pattern of the vasculature of the avian foot was called a (mainly) dorsal supply [21,27]. However, the me_DA1 arose from the prominent arterial plantar arch in the northern goshawk, the common buzzard, and the barn owl [27]. This pattern was referred to as a partly plantar supply of the avian foot [21,27]. This partly plantar supply was also observed in the domestic chicken, with the difference that not only the me_DA1 but also the common trunk for the la_DA2 and me_DA3 originated from the arterial plantar arch [40,54]. Ducks and geese showed an entirely [21] or—similar to the domestic chicken—at least a partly [55,56,57] plantar supply of the feet. El Nahla [58] described that in the ostrich (*Struthio camelus*), the pulvinar artery originated from the common dorsal metatarsal artery in the region of the metatarsophalangeal joints and ran through the distal metatarsal foramen to the plantar side of the foot. This could mean that also in the ostrich, the supply of the metatarsal pad is from the plantar side via the arterial plantar arch. In a multi-species description by Baumel [59], it is merely stated that pulvinar arteries arose from the common dorsal metatarsal artery in the region of the metatarsophalangeal joints and supplied the metatarsal pad possibly indicating a dorsal supply. It remains speculative whether these variations affect the blood supply of the metatarsal pad. In conclusion, the differences associated with the dorsal or plantar supply of the foot may influence the vascularization of the metatarsal foot pad.

For the drainage of the metatarsal pad, we found several digital veins, which received pulvinar branches in the examined species of birds of prey and owls. We did not find any description of pulvinar veins in previous studies on birds of prey and owls. Vollmerhaus and Hegner [40] gave a detailed description of pulvinar veins in the domestic chicken. Our results were largely similar to their findings and in both studies pulvinar veins drained into the following digital veins: the la_DV2, the me_DV2, the la_DV3, the me_DV3, and the la_DV4 as well as the me_DV4. In contrast to our findings, Vollmerhaus and Hegner [40] did not mention the la_DV1 as a possible junction for a venous pulvinar branch in the domestic chicken and mentioned not only one, but several pulvinar branches joining into the la_DV4. Furthermore, none of the described venous drainages of the metatarsal pad were explicitly identified as the main drainage in the chicken [40]. For all birds of prey and owls examined in the present study, we revealed a main drainage of the metatarsal pad, which was positioned on the lateral side of the foot—opposed to the main arterial supply on the medial side, which was positioned on the medial side. The main drainage might be on the lateral side because the la_DV4 and the la_DV1, both large digital veins, joined here forming a large common digital vein on the plantar side of the foot (lateral plantar common digital vein) [27]. The other two large digital veins, which received pulvinar venous branches, joined common digital veins at the dorsal side of the foot [27], which might be less favorable for the blood flow from the metatarsal pad. On the contrary, the large me_DA1 supplying the plantar-facing first toe is most convenient for the origin of the main pulvinar artery. In this way, a similar vascular pattern seems to emerge for the arteries and veins of the metatarsal pad as has already been observed for the arteries and veins on the lateral and medial side of the toes, whose diameter showed a reciprocal asymmetry in their size [21,27].

For the blood vessel supply of the digital pads, our results showed brace-like branches arising from the digital arteries and, respectively, draining into the digital veins. These brace-like branches ramified in the skin of the digital pads at the plantar side of the foot, which is in accordance to the findings in the domestic chicken [40].

## 5. Conclusions

Summarizing the results of the study, no species-specific variations were detected in the structure of the epidermis and dermis as well as in the pattern of the dermal vascular networks. The main difference we found between the species studied was the presence and amount of fat tissue in the subcutis as the basis for the metatarsal foot pad: While the examined falcon and owl species showed organized fat tissue bodies, in northern goshawks and common buzzards, the subcutis consisted mainly of connective tissue. Due to the organized fat tissue body occupying space in the subcutis, the metatarsal pad was more protruding in falcons and owls and rather flat in the northern goshawk and the common buzzard. This resulted in species-specific differences in the course of the main pulvinar artery supplying the metatarsal pad. These results can be discussed with regard to the development and the species-specific susceptibility of bumblefoot in birds of prey and owls.

In the literature, pododermatitis is described to occur at the plantar surface of the foot [1,2,14]; some authors go into more detail and define specific localizations as the metatarsal pad [2,3,14,20]. Photographs of bumblefoot lesions shown by these authors have in common that, in particular, the transition area between the metatarsal pad and the area cranial to the metatarsal pad is affected in the disease’s early stages [2,3,14], which is in accordance to our own clinical experiences. The results of the present study showed that, in all species examined, the metatarsal pad had more prominent dermal and corresponding epidermal papillae and well-developed dermal and subdermal vascular networks and, in some species, an organized fat tissue pad—adaptations to mechanical stress—in contrast to the less-well-protected interpulvinar area cranial to the metatarsal pad. In consequence, unsuitable perching surfaces or a swelling of the foot due to an edema or an inflammation could bring the area cranial to the metatarsal pad, which is anatomically not adapted for a high mechanical stress, more into strain. Therefore, the area cranial to the metatarsal pad could be particularly susceptible to ischemic pressure necrosis and might be predisposed to the development of bumblefoot lesions in the case of non-physiological, mechanical stress.

The main function of the metatarsal fat pad is to protect vulnerable subcutaneous structures from pressure and injury [14,40,46]. It could be assumed that an overload or permanent pathological pressure load due to suboptimal husbandry conditions, such as the birds being overweight, a lack of exercise, or inappropriate perching surfaces [2,14,15,16], might lead to a deformation or disalignment of the fat pad and result in a loss of its function and an increased load on other areas as the interpulvinar area cranial to the metatarsal pad. We revealed species-specific variations in the course of the main arterial pulvinar branch due to the characteristic of the metatarsal fat pad. Vascular resistance depends not only on the diameter but also on the length of a vessel according to the Hagen–Poiseuille equation. Therefore, a better blood flow to the skin of the metatarsal pad in the species without an organized fat tissue body, which show a straighter and, therefore, also shorter course before ramifying into the skin, can be assumed (Hagen–Poiseuille equation). In falcons and owls, a fat pad disalignment or deformation might also bring the risk of the compression of pulvinar arteries vessels encircling the fat pad, which could impair the blood flow to the skin on the plantar aspect of the metatarsal pad. In addition, the degree of the organization of the fat pad could play a role in the ability to withstand mechanical stress: A better partitioned metatarsal pad by connective tissue septa as in the examined owls might withstand deformations caused by pressure overload better than a non-partitioned as in the examined falcons. In conclusion, pathological mechanical stress might more likely overstrain the fat pad in falcons and owls than the flatter connective tissue pad in goshawks and buzzards.

Once there is a pressure necrosis of the skin, pathogens might overcome the damaged skin barrier more easily causing a secondary bacterial infection of the underlying structures [60]. In this case, a large fat pad as the basis for the metatarsal pad in falcons and owls might promote the spread of pathogens due to the absence of larger blood vessels, potentially hindering healing and supporting the development of bumblefoot. The flexor tendons pass the metatarsophalangeal joints at the plantar side of the foot directly underneath the metatarsal pad [2,24]. In this area, hardly any supplying blood vessels were visible around the flexor tendons in the present study, which was in accordance with Harcourt-Brown [2,24]. The spread of secondary bacterial infections in the area of the flexor tendons might be facilitated by the fact that they are poorly vascularized [61]. We hypothesize that northern goshawks and common buzzards use a foot arch as a pressure distribution mechanism of the foot sole. This would differentiate them from falcons and owls, which rely on a fat pad as the basis for the metatarsal pad to distribute pressure and protect against pressure necrosis.

It is assumed that the pathogenesis of bumblefoot is connected with the development of cardiovascular problems causing edema in the feet when birds abruptly reduce their activity level [3,17]. This discrepancy of activity levels could be of greater consequence for falcons as they are “long-distance-hunters” in comparison to goshawks and buzzards and might, therefore, be more vulnerable to circulatory disorders of the feet [3,17].

All aspects mentioned could be involved in the fact that falcons show a higher susceptibility for the development of bumblefoot than goshawks and buzzards. Owls might occupy a middle position. Unlike goshawks and buzzards, they might rely on their metatarsal fat pad to distribute pressure, but, compared to falcons, their degree of “adaptive specialization for prolonged locomotor activity” appears to be lower, as indicated by a smaller heart muscle mass [62]. In conclusion, biological differences between species in flight activity and hunting behavior [63,64] could help to explain why hawks, buzzards, and owls are less vulnerable to cardiovascular disorders than falcons.

In addition, the entire vascular supply of the feet must be taken into account when evaluating the etiology and species-specific susceptibility for the development of bumblefoot. In this context, especially the species-specific variations in the strength of the arterial plantar arch should be taken into account. The arterial plantar arch was located at the level of the metatarsophalangeal joints deep underneath the flexor tendons in the region of the metatarsal pad in all eight examined species [27]. Due to their partly plantar supply of the foot, it was more prominent in northern goshawks and common buzzards (Accipitriformes) than in the examined falcons (Falconiformes) and owls (Strigiformes), which had a mainly dorsal supply [27]. In our former study, we assumed that this could result in a better vascular supply of this region in goshawks and buzzards than in falcons and owls [27]. However, we did not find remarkably more or particularly stronger pulvinar branches arising from the plantar arch and supplying the metatarsal pad in northern goshawks and common buzzards than in the falcon and owl species examined.

The occurrence and distribution of pulvinar arteries and veins did show minor individual differences (see Table 3 and Table 4). Thus, the question arises whether these variations in the vascularization of the metatarsal pad might be even responsible for individual prevalence for the development of bumblefoot.

Pododermatitis often requires surgical treatment [1,2]. As circulatory disorders are discussed as etiology for the development of pododermatitis [2,3,5], it is particularly important to spare vessels during surgery on the feet. The present study visualized the topography of pulvinar vessels and will, therefore, help to better protect vessels during surgical procedures on the foot sole in birds of prey and owls.

## Figures and Tables

**Table 1:** Number of examined specimens per species, sex, and research method (dissection after injection with latex (Latex), contrast µCT scans (µCT), histology, and corrosion casts).

	Latex			µCT			Histology			Corrosion Casts			Total
	Total	Male	Female	Total	Male	Female	Total	Male	Female	Total	Male	Female	
Northern goshawk	5	2	3	3	1	2	6	3	3	8	4	4	22
Common buzzard	7	4	3	3	1	2	6	3	3	8	2	6	24
Peregrine falcon	4	2	2	4	1	3	6	6	0	9	6	3	23
Gyr–saker falcon	5	5	0	3	3	0	6	6	0	5	5	0	19
Common kestrel	3	2	1	3	2	1	6	3	3	9	3	6	21
Eurasian eagle-owl	4	2	2	4	2	2	6	2	4	6	4	2	20
Long-eared owl	3	2	1	3	2	1	6	3	3	7	4	3	19
Barn owl	4	3	1	3	2	1	6	3	3	8	4	4	21

**Table 3:** Examination of the arterial pulvinar branches supplying the metatarsal foot pad: number of feet with visible arterial pulvinar branch per avian species and investigation method (dissection after injection of latex (Latex) and contrast µCT scans (µCT)) in relation to the total number of feet examined.

	Northern Goshawk		Common Buzzard		Peregrine Falcon		Gyr–Saker Falcon		Common Kestrel		Eurasian Eagle-Owl		Long-Eared Owl		Barn Owl	
	Latex	µCT	Latex	µCT	Latex	µCT	Latex	µCT	Latex	µCT	Latex	µCT	Latex	µCT	Latex	µCT
Total number of feet examined for pulvinar arteries	3	6	4	6	3	7	3	6	3	6	3	7	3	6	3	6
Arterial pulvinar branch arising from…																
…medial 1st toe (me_DA1)	3	6	4	6	3	7	3	6	3	4	1	7	3	6	3	6
…medial 2nd toe (me_DA2)	0	0	0	0	0	0	0	0	0	0	2	2	0	1	0	0
…lateral 2nd toe (la_DA2)	3	5	4	6	1	7	3	6	2	0	3	7	2	6	1	5
…split point into la_DA2/me_DA3	0	0	0	0	0	0	1	0	0	0	0	0	0	0	0	0
…medial 3rd toe (me_DA3)	0	0	0	0	0	0	0	0	0	0	0	5	0	2	2	5
…lateral 3rd toe (la_DA3)	1	1	0	2	0	2	0	0	0	0	0	2	0	1	0	0
…split point la_DA3/me_DA4	1	2	1	3	0	0	0	0	0	0	1	0	1	0	0	0
…medial 4th toe (me_DA4)	1	2	3	1	3	5	2	5	1	0	2	7	2	6	2	6
…lateral 4th toe (la_DA4)	0	0	0	0	0	0	0	0	0	0	0	1	0	0	0	0
…arterial plantar arch	2	0	3	0	1	0	2	0	0	0	2	0	0	0	0	0

**Table 4:** Examination of the venous pulvinar branches draining the metatarsal foot pad: number of feet with visible venous pulvinar branch per avian species and investigation method (dissection after the injection of latex (Latex) and µCT scans (µCT)) in relation to the total number of feet examined.

	Northern Goshawk		Common Buzzard		Peregrine Falcon		Gyr–saker Falcon		Common Kestrel		Eurasian Eagle-Owl		Long-Eared Owl		Barn Owl	
	Latex	µCT	Latex	µCT	Latex	µCT	Latex	µCT	Latex	µCT	Latex	µCT	Latex	µCT	Latex	µCT
Total number of feet examined for pulvinar veins	3	6	4	6	3	7	4	6	1	6	4	7	3	6	3	6
Venous pulvinar branch joining into…																
…lateral 1st toe (la_DV1)	2	6	4	4	0	5	1	3	1	1	1	2	1	0	0	3
…lateral 4th toe (la_DV4)	1	2	0	0	1	3	1	2	0	0	2	3	2	3	0	0
…medial 4th toe (me_DV4)	0	1	0	0	0	0	1	0	0	0	2	2	1	0	1	0
…split point me_DV4/la_DV3	0	0	0	2	0	0	0	0	0	0	1	1	0	0	0	0
…lateral 3rd toe (la_DV3)	0	0	0	0	1	0	0	0	0	0	0	1	0	0	0	0
…medial 3rd toe (me_DV3)	2	3	1	0	0	0	0	0	0	0	3	3	0	0	0	0
…split point me_DV3/la_DV2	0	0	0	1	0	0	0	0	0	0	0	0	0	0	0	0
…lateral 2nd toe (la_DV2)	0	0	0	0	1	1	3	0	0	0	0	0	0	0	1	0
…medial 2nd toe (me_DV2)	2	2	2	1	0	0	1	1	1	0	2	0	1	0	0	0
…medial 1st toe (me_DV1)	0	1	0	3	0	0	0	0	0	0	0	0	0	0	0	0
…venous plantar arch	0	0	0	0	1	0	2	0	0	0	4	0	0	0	0	0

## Data Availability

Data are contained within the article.
